# Fate of the H-NS–Repressed *bgl* Operon in Evolution of *Escherichia coli*

**DOI:** 10.1371/journal.pgen.1000405

**Published:** 2009-03-06

**Authors:** T. Sabari Sankar, Girish Neelakanta, Vartul Sangal, Georg Plum, Mark Achtman, Karin Schnetz

**Affiliations:** 1 Institute for Genetics, University of Cologne, Cologne, Germany; 2 Department of Molecular Biology, Max-Planck Institute for Infection Biology, Berlin, Germany; 3 Department of Microbiology and Environmental Research Institute, University College Cork, Cork, Ireland; 4 Institute for Medical Microbiology, Immunology, and Hygiene, University of Cologne, Cologne, Germany; Universidad de Sevilla, Spain

## Abstract

In the enterobacterial species *Escherichia coli* and *Salmonella enterica*, expression of horizontally acquired genes with a higher than average AT content is repressed by the nucleoid-associated protein H-NS. A classical example of an H-NS–repressed locus is the *bgl* (aryl-β,D-glucoside) operon of *E. coli*. This locus is “cryptic,” as no laboratory growth conditions are known to relieve repression of *bgl* by H-NS in *E. coli* K12. However, repression can be relieved by spontaneous mutations. Here, we investigated the phylogeny of the *bgl* operon. Typing of *bgl* in a representative collection of *E. coli* demonstrated that it evolved clonally and that it is present in strains of the phylogenetic groups A, B1, and B2, while it is presumably replaced by a cluster of ORFans in the phylogenetic group D. Interestingly, the *bgl* operon is mutated in 20% of the strains of phylogenetic groups A and B1, suggesting erosion of *bgl* in these groups. However, *bgl* is functional in almost all B2 isolates and, in approximately 50% of them, it is weakly expressed at laboratory growth conditions. Homologs of *bgl* genes exist in *Klebsiella*, *Enterobacter*, and *Erwinia* species and also in low GC-content Gram-positive bacteria, while absent in *E. albertii* and *Salmonella* sp. This suggests horizontal transfer of *bgl* genes to an ancestral Enterobacterium. Conservation and weak expression of *bgl* in isolates of phylogenetic group B2 may indicate a functional role of *bgl* in extraintestinal pathogenic *E. coli*.

## Author Summary

Horizontal gene transfer, an important mechanism in bacterial adaptation and evolution, requires mechanisms to avoid uncontrolled and possibly disadvantageous expression of the transferred genes. Recently, it was shown that the protein H-NS selectively silences genes gained by horizontal transfer in enteric bacteria. Regulated expression of these genes can then evolve and be integrated into the regulatory network of the new host. Our analysis of the catabolic *bgl* (aryl-β,D-glucoside) operon, which is silenced by H-NS in *E. coli*, provides a snapshot on the evolution of such a locus. Genes of the *bgl* operon were presumably gained by horizontal transfer from Gram-positive bacteria to ancestral enteric bacteria. In *E. coli*, the *bgl* operon co-evolved with the diversification of the species into four phylogenetic groups. In one phylogenetic group the *bgl* operon is functional. However, in two other phylogenetic groups, *bgl* accumulates disrupting mutations, and it is absent in the fourth group. This indicates that the H-NS–silenced *bgl* operon evolved differently in *E. coli* and is presumably positively selected in one phylogenetic group, while it is neutrally or negatively selected in the other groups.

## Introduction

The species *Escherichia coli* includes commensal strains residing in the intestine of humans and animals, as well as pathogenic strains causing various intestinal and extra-intestinal infections. This diversity in the life-style of *E. coli* is based on a significant genetic variability of their genomes. Sequencing of *E. coli* genomes including that of the laboratory strain K12 (MG1655), the uropathogenic (UPEC) strain CFT073, and the enterohaemorrhagic (EHEC) strains O157∶H7 EDL933 and Sakai, demonstrated that the *E. coli* genome, like that of other bacteria, consists of a conserved core genome and a variable pool of genes [1]–[4]. Genes of the core genome are present in all *E. coli* isolates, while variable genes are interspersed in the core genome as genomic islands (also named islets or loops) and only present in a subgroup of strains or in single isolates [2]–[4]. The extensive difference in the gene content of bacterial genomes is caused by horizontal gene transfer and gene loss, which contribute dominantly to bacterial evolution, as evident for the evolution of γ-proteobacteria and for the diversification of *E. coli* [5],[6]. Furthermore, the species *E. coli* is subdivided into four phylogenetic groups (A, B1, B2, and D). These groups were initially detected by multi locus enzyme electrophoresis (MLEE), and are also reflected by multi locus sequence typing (MLST) [7]–[9]. Furthermore, MLST typing demonstrated frequent recombination of strains of different phylogenetic groups resulting in hybrid strains (AxB1 and ABD) [8]. Genome and phylogenetic analysis also demonstrated that *Shigella* strains belong to the species *E. coli* [5],[10]. In addition, *E. coli* strains have been identified, which form a second population distinct from the main *E. coli* population with its 4 phylogenetic groups. These rare strains presumably represent descendents of a subpopulation that diverged early in evolution of *E. coli*, prior to the generation of the 4 ‘modern’ phylogenetic groups A, B1, B2, and D [8],[11].

**Figure 1:**
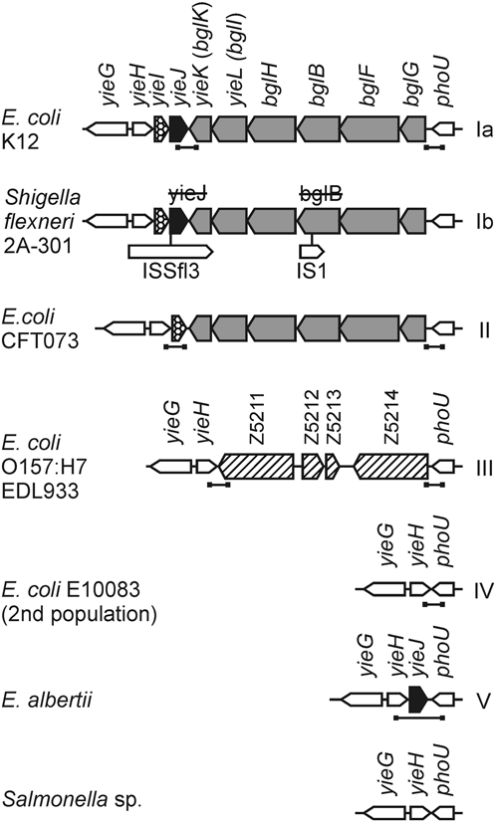
Structure of the *yieH*-*phoU* region in *E. coli*, *E. albertii*, and *Salmonella* sp. The *bgl* operon maps next to *phoU* in *E. coli* K12, in *Shigella flexneri*, and in *E. coli* CFT073. Genes *bglG*, *bglF*, and *bglB* are necessary and sufficient for the uptake and hydrolysis of aryl-β,D-glucosides such as salicin and arbutin [29],[64],[65]. Gene *bglG* encodes the positive regulator and antiterminator BglG, *bglF* encodes the β-glucoside specific permease EII^Bgl^, and *bglB* encodes the phospho-β,D-glucosidase BglB. The fourth gene *bglH* (*yieC*) encodes a porin specific for the uptake of aryl-β,D-glucosides [66]. Genes *yieL* (*bglI*) and *yieK* (*bglK*) which are presumably part of the *bgl* operon encode putative proteins homologous to endo-1-4-xylanase and glucosamine-6-phosphate-isomerase/deaminase, respectively. Further, gene *yieJ* is present only in *E. coli* K12, *S. flexneri* and *E. albertii*, while the *yieI* gene is present in *E. coli* K12, *S. flexneri*, and CFT073 but not in *E. coli* O157:H7 (Figure 1). In *S. flexneri* the *bgl* operon is disrupted by insertion element IS1. In all sequenced *Salmonella* strains of the species *S. enterica* and *S. bongori* the structure of the chromosomal region is similar as shown. The location of PCR fragments used for typing and sequencing is indicated by horizontal bars underneath the schemes. The types Ia, Ib, II, III, IV, and V which were assigned to the different loci are indicated to the right of the schemes.

Among the variable gene pool of *E. coli*, pathogenicity islands have been best characterized and they provide models for the locus specific analysis of genome evolution by horizontal gene transfer [12]. Less is known about genomic islands which encode gene products not apparently related to pathogenicity and genes of unknown function. A locus of this type maps next to the *E. coli pst-phoU* operon [1], where two alternative islands (or islets) exist. In the laboratory strain K12 and the UPEC strain CFT073 an island is present which carries the *bgl* operon encoding the gene products for uptake and hydrolysis of aryl-β,D-glucosides (Figure 1). In *E. coli* O157∶H7 EDL933 another island of four open reading frames of unknown function (Z5211 to Z5214) is present instead of the *bgl* locus (Figure 1). The Z5211 to Z5214 open reading frames represent ORFans with no close homologs in any other genome which are sequenced up to date [5].

Our interest in the *E. coli bgl* locus is based on the finding that the operon is silent (‘cryptic’) [13]–[16]. The *bgl* operon is repressed by the nucleoid-associated protein H-NS, a global regulator and ‘genome sentinel’ [17],[18], and for *E. coli* K12 no laboratory growth conditions are known allowing its expression [14], [19]–[21]. Silencing of the *bgl* operon by H-NS can be overcome and the operon can be ‘activated’ by mutation of the *hns* gene or by mutations that interfere with repression by H-NS [21]–[25]. The latter includes mutations causing constitutive expression of *bglJ* and *leuO*, respectively. LeuO and BglJ are positive regulators, which presumably bind next to the *bgl* promoter and counteract repression by H-NS [24]. In addition, mutations mapping *in cis* to the *bgl* promoter occur, which include integration of insertion elements, deletions within the H-NS binding region, and point mutations which improve the binding site for the cAMP-dependent regulator protein (CRP) [20]. Once ‘activated’, the *bgl* operon becomes inducible by substrate demonstrating that it is maintained in a functional but silent state in *E. coli* K12 [13],[14]. However, up to date the biological significance of silencing of the *bgl* operon has remained puzzling. Early, it was speculated that the *bgl* operon may be cryptic because of the abundance of cyanogenic β,D-glucosides in nature, whose hydrolysis by the operon encoded phospho-β,D-glucosidase BglB would release the toxic aglycon, and that mutational activation of *bgl* in some cells might provide a selective advantage for the population at certain conditions [14]. Then, it turned out that the sugar-specific control of the *bgl* operon by transcriptional antitermination, and the control of the activity of the operon-encoded specific antiterminator protein, BglG, by the PTS (phosphoenolpyruvate-dependent phosphotransferase system) is a regulatory mechanism typical of low GC-content Gram-positive bacteria [26],[27]. The further findings that the codon usage of *bgl* is atypical for *E. coli* but similar to *Bacillus subtilis*, and that the activity of BglG, in contrast to that of other PTS-regulated proteins in *E. coli*, can be well controlled by PTS proteins from *Bacillus subtilis* [28],[29], may suggest that the *bgl* operon originates from a horizontal transfer event from low GC-content Gram-positive bacteria. Repression of *bgl* by H-NS may also support the idea that the *bgl* locus was horizontally transferred to *E. coli*. H-NS prevents the un-controlled expression of horizontally transferred AT-rich DNA including that of pathogenicity islands in *E. coli* and *Salmonella enterica* [17], [18], [30]–[32]. In general, repression by H-NS can be relieved by binding of specific transcription factors and changes in the local DNA conformation, processes which depend on specific environmental stimuli [18],[33]. Several H-NS repressed loci are expressed in the host-environment only [18],[33], and *bgl* operon expression was detected in a septicemic *E. coli* isolate when infecting mouse liver indicative of a role of the *bgl* operon in the host [34].

To characterize the fate of the *bgl* operon in evolution, we typed the chromosomal locus by PCR and sequencing in a collection of 174 strains, comprised of 171 *E. coli* isolates including strains of the ECOR collection [35], and 3 representatives of the closely related species *Escherichia albertii* [8],[36]. Different types of the locus were identified by this approach and their clonal divergence in *E. coli* was traced by mapping onto a minimal spanning tree representing the clonal structure of the strain collection. In addition, all the strains were analyzed for their aryl-β,D-glucoside phenotype, and the phenotypes were likewise correlated with the clonal structure of the collection. These analyses demonstrated clonal inheritance of *bgl* in *E. coli* and further phylogenetic analyses suggest that the *bgl* operon originates from a horizontal transfer event from low GC-content Gram-positive bacteria to an ancestral Enterobacterium. Weak expression of *bgl* in strains of the phylogenetic group B2 of *E. coli* may indicate a functional role of *bgl* in an ecological niche occupied by extra-intestinal pathogenic *E. coli* (ExPEC).

## Results

### Phylogeny and Population Structure of the *E. coli* Collection

For the analysis of the evolution of the *bgl* operon in *E. coli* we chose a collection of *E. coli* strains which includes the ECOR reference strains [35], as well as 96 human *E. coli* strains isolated in the local medical microbiological diagnostic center. These latter strains include 51 commensals isolated from healthy humans, as well as 24 septicemic and 21 uropathogenic isolates (Table S1). In addition, two uropathogenic strains J96 and 536 [37] were analyzed, as well as the septicemic strain i484 in which expression of *bgl* upon infection of mouse liver was shown [34]. Furthermore, two *E. coli* strains (RL325/96 and Z205) of a second *E. coli* population, presumably representing descendents of a subpopulation that diverged early in evolution of *E. coli*, and three representatives of the closely related species *Escherichia albertii* were included in the analysis [8].

**Figure 2:**
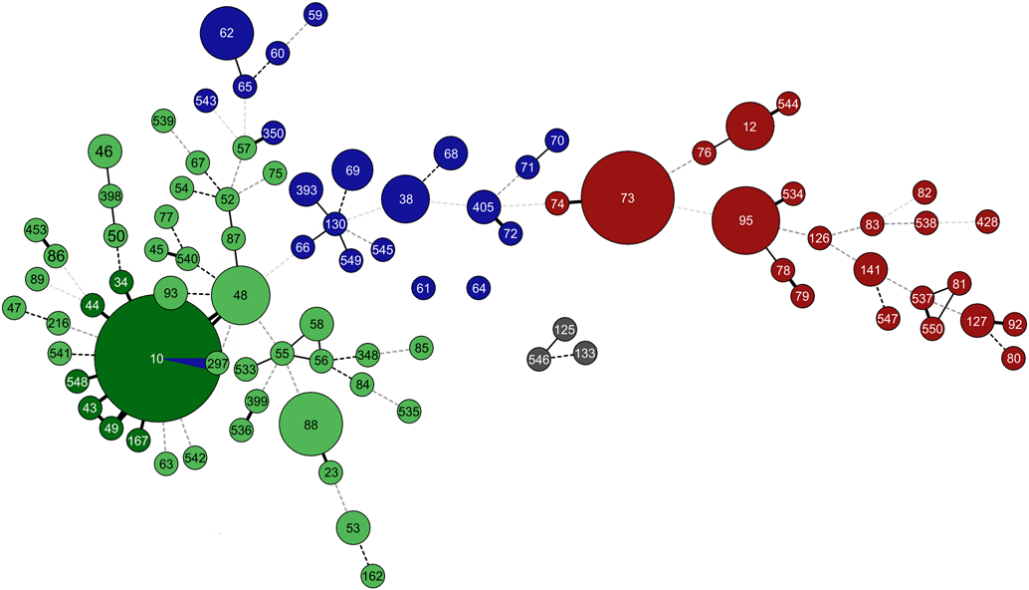
Distribution of *bgl-Z* locus types within a minimal spanning tree of 174 *E. coli* strains. The types of the *bgl*-Z locus are color encoded. Type Ia shown in dark green maps to sequence types (STs) belonging to the ST10 complex and includes only strains of the phylogenetic group A; type Ib shown in light green maps to strains belonging to the phylogenetic groups B1 and A, as well as AxB1 and ABD hybrid strains; type II shown in red maps to STs which all belong to the phylogenetic group B2; type III shown in blue maps to STs which belong to the phylogenetic group D and to ABD hybrid strains, and type IV shown in grey maps to strains representing a second population of *E. coli* which presumably diverged early in evolution of *E. coli*. The sequence types (ST) are indicated and the size of the circle correlates with the number of strains by which an ST is represented.

The population structure of this collection was established by multi locus sequence typing (MLST), as described [8] (for details see Materials and Methods), and is visualized by a minimal spanning tree (MS_TREE_) (Figure 2). The strain collection represents 91 sequence types (STs) and 25 ST complexes, and thus is representing the *E. coli* diversity. Interestingly, one of the human commensal isolates, E10083 (sequence type ST546), mapped next to the two strains RL325/96 (ST133) and Z205 (ST125) of the second *E. coli* population, which were isolated from dog and parrot, respectively [8] (shown in grey in Figure 2). This suggests that the human isolate E10083 is probably another representative of the second population of *E. coli*, which presumably diverged early in evolution of *E. coli* prior to formation of the 4 phylogenetic groups A, B1, B2, and D [8].

Further, the concatenated sequences of the 7 MLST loci of each strain were used for phylogenetic analysis by construction of neighbor-joining (NJ) trees (Figure S1). As a reference, the sequences of the MLST loci extracted from published *E. coli* genome sequences (Table S2) were included. The structure of the NJ tree of the local isolates was very similar to that of the ECOR strains (Figure S1, compare A and B). Four major clades representing the known phylogenetic groups A, B1, B2 and D of *E. coli* were apparent [7],[38] (Figure S1). Again, strain E10083 from the collection of local isolates diverged from the four major clades and clustered with the strains RL325/96 and Z205 representing the second population of *E. coli* [8] (Figure S1). Furthermore, for each isolate the phylogenetic group was either extracted from the MLST database or determined, as described [8] (listed in Table S1). In total, 48 strains of the collection belong to the phylogenetic group A, 17 strains to B1, 48 to B2, and 21 to D, while 16 strains belong to hybrid group AxB1 and 13 to ABD. For 5 strains the assignment was ambiguous, and 3 strains represent the second *E. coli* population (Table S1). The NJ trees and the representation of the phylogenetic groups likewise suggest that the strain collection is representing the diversity of *E. coli*.

### Typing of the *bgl* Locus

For typing the chromosomal locus at which *bgl* is located, we first analyzed by PCR whether the *bgl* operon is present, whether it is replaced by a cluster of 4 genes (Z5211 to Z5214, in the following named Z-locus) as in the O157∶H7 strains (Figure 1), or whether the locus has another structure. The PCR revealed that 77% of the strains (135 of 174) carry the *bgl* operon and that 19% of the strains (33 of 174) carry the Z-locus. The three strains representing the second population of *E. coli* and the three *E. albertii* strains carried neither the *bgl* nor the Z locus (Figure 1). The PCR analysis further demonstrated that in strains that carry the *bgl* operon two variants exist: in some strains the structure is similar to the one in K12 with two genes *yieI* (*cbrB*) and *yieJ* (*cbrC*) present downstream of *bgl*, while in other strains the structure is similar to the *bgl* locus in CFT073, where only the *yieI* (*cbrB*) gene is present downstream of *bgl* (Figure 1). In several strains the *bgl*-*yieIJ* locus and the Z-locus, respectively, carried deletions and/or were disrupted by insertion elements. These mutations were characterized in detail by PCR and sequencing, and the results are summarized in the supplement (Figure S2). Furthermore, Southern blots were performed of all locally isolated strains which did not carry *bgl* at its normal locus (Figure S3 and Table S1). The Southern blots performed with probes for the *bglG*-*bglF* genes (Figure S3) and for all other genes present in the *bgl-yieIJ* locus (not shown) demonstrated that these strains do not carry the *bgl* genes elsewhere in the genome. This analysis included 17 strains which carried a Z locus and also strain E10083, the representative of the second population of *E. coli*, which does neither carry *bgl* nor the Z locus (Figure S3, Table S1, and [39]).

In a second step of typing, fragments encompassing the ends of the *bgl* and the Z gene cluster, respectively, were sequenced to examine the diversity of these loci (Figure 1). For strains which carry the *bgl* island, the sequence of 534 bp derived from the left end and 277 bp derived from the right end of the island were concatenated. These 811 bp sequences were aligned and used for construction of NJ trees (Figure S4). Strains, in which the analyzed region of the *bgl* locus was disrupted by insertions and deletions, were omitted. Separate NJ trees were generated for ECOR strains and the remaining strains, and sequences derived from published *E. coli* genomes were included in both trees (Figure S4). The sequences clustered into three major clades, demonstrating the presence of three types of the *bgl* locus in modern *E. coli* isolates which were designated as types Ia, Ib, and II. The Z5211-5214 locus was analyzed similarly, but no sub-types were assigned because of a high degree of sequence variations and limiting number of sequences (not shown). Thus, all strains harboring the Z5211-5214 locus were assigned as type III. The strains of the second *E. coli* population and the *E. albertii* strains lack the *bgl* operon and the Z5211-5214 locus, as analyzed by PCR and sequencing (Figure 1). The sequences of the three strains of the second *E. coli* population, including E10083 isolated here, are identical with only one SNP (single nucleotide polymorphism). These strains were assigned as *bgl*-Z locus type IV. As stated above, the presence of *bgl* genes in strain E10083 was excluded by Southern analysis. The *E. albertii* strains carry the *yieJ* (*cbrC*) gene next to *phoU* and were assigned as locus type V (Figure 1). Further, no homologs to the genes of the *bgl* and Z loci were found by TBLASTN on the genome sequence of *E. albertii* (Table S2) indicating the absence of *bgl* and Z genes in *E. albertii* (data not shown).

### The *bgl* and Z Islands Evolved Clonally

To analyze the diversification of the *bgl* and Z loci in relation to the clonal structure of the *E. coli* collection, the *bgl*-Z-locus types Ia, Ib, II, III, and IV were mapped color-coded onto the minimal spanning tree (MS_TREE_) (Figure 2). This revealed a strong correlation of the structure and diversification of the *bgl*-Z-locus with the clonal structure of the population. All *bgl* type Ia strains (shown in dark green) mapped to the ST10 complex, and all these strains belong to the phylogenetic group A. The type Ib strains (shown in light green) mapped to several ST complexes, which mainly represent strains of the phylogenetic group B1, as well as AxB1 and ABD hybrid strains, and some A strains. The *bgl* type II (shown in red) mapped to the ST73, ST95 and ST12 complexes as well as related STs, which all belong to the phylogenetic group B2. The *bgl*-Z-locus type III strains (which carry the Z5211-5214 gene cluster, shown in blue) mapped to different ST complexes, like ST31, ST38, and ST59, which represent the phylogenetic group D or ABD hybrid strains. Importantly, there is only one case indicative of a recombination event: Strain F905, a ST10 strain belonging the phylogenetic group A carries a Z5211-Z5214 gene cluster instead of a *bgl* type Ia locus. Taken together, these analyses revealed a strong congruence of evolution of the *bgl*-Z-locus with the species.

### Phylogenetic Analysis of the Complete *bgl* Island

**Figure 3:**
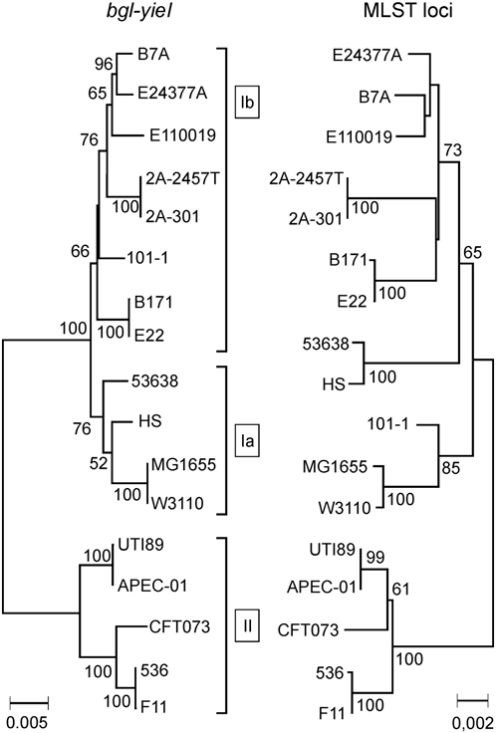
Congruence of the phylogeny of the *bgl* operon with the *E. coli* phylogeny. The sequence of the *bgl* operon was extracted from the genome sequences of the strains indicated, aligned and analyzed by a NJ tree. For comparison the sequences of the seven MLST loci were also extracted from the genome sequences, concatenated and similarly used for NJ construction. The types of the *bgl* operon are indicated by Ib, Ia, and II. The analysis of the topological similarity between the trees [67] gave a index of congruence with a significant P-value (0.00026).

Typing of the *bgl* locus based on sequences of small fragments revealed a strong congruence with the clonal structure and phylogeny of *E. coli*. To further analyze the correlation of the phylogeny of *bgl* with that of the species, the sequence of the entire *bgl*-locus including genes *bglG*, *bglF*, *bglB*, *bglH*, *bglI* (*yieL*), and *bglK* (*yieK*) as well as the downstream genes *yieJ* and *yieI* (*crbBC*), was extracted from the genome sequences of 17 *E. coli* and *Shigella* strains (Table S2). Of these sequences a multiple alignment was generated and a NJ tree was constructed (Figure 3). In some of these strains (including *S. flexneri* strains 2A-301 and 2457T, as well as the *E. coli* strains E22, E110019, and 53638) insertion elements map within the *bgl* locus. The sequences of these insertion elements were manually removed to allow alignment. The NJ tree again clustered into 3 clades (Figure 3) and was very similar to the NJ tree which was based on sequence fragments of the *bgl* locus (Figure S4). To correlate the phylogeny of the whole locus with the species phylogeny, the sequences of the seven MLST loci were also extracted from the 17 genome sequences, concatenated and used for construction of a NJ tree (Figure 3). The comparison of the NJ tree based on the *bgl* locus with the NJ tree based on the MLST loci revealed a strong congruence with minor deviations (Figure 3), in agreement with the results shown above (Figure 2). A major incongruence concerned strain 101-1, which indicates a recombination event.

### Expression and Functionality of the *bgl* Operon

**Figure 4:**
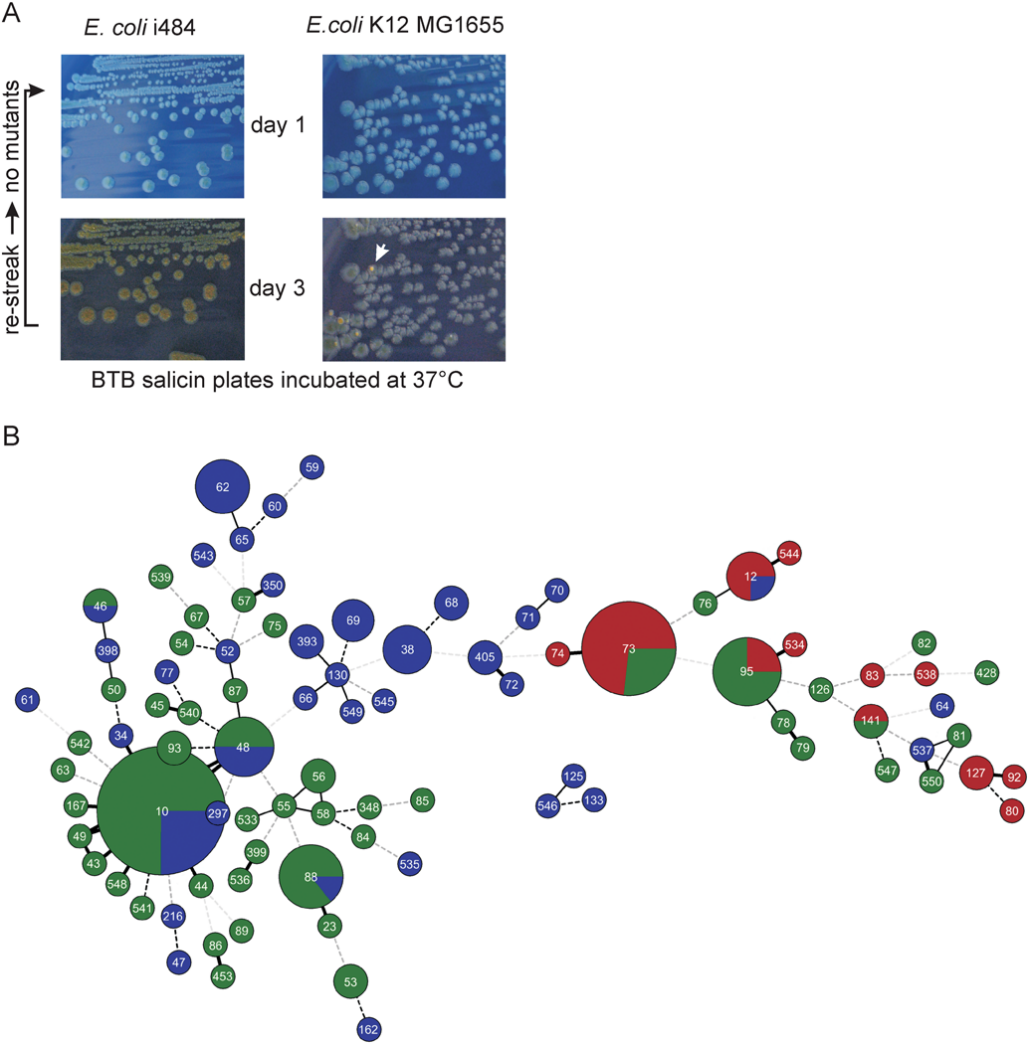
Expression of the *bgl* operon. (A) Bgl phenotype of i484 on salicin indicator plates after 1 and 3 days of incubation at 37°C in comparison to the phenotype of *E. coli* K12 strain MG1655. The arrow indicates a Bgl-positive mutant of strain MG1655 which appears as papillae. Re-streaking of weakly positive colonies of strain i484 demonstrated that they are no mutants. (B) Distribution of the Bgl phenotype within a minimal spanning tree. The Bgl phenotypes were mapped color-coded on the MS_TREE_ with red for weakly Bgl-positive strains, green for strains carrying a silent but functional operon, and blue for Bgl-negative strains.

In *Escherichia coli* K12 expression of the *bgl* operon is repressed by H-NS under all laboratory growth conditions tested so far. However, spontaneous Bgl-positive mutants, in which repression of *bgl* by H-NS is relieved, can be easily isolated on salicin indicator plates, where the mutants appear as papillae. The spectrum of mutations which relieve repression include point mutations and small deletions mapping *in cis* to the *bgl* promoter. These mutations are expected to occur in all strains, in contrast to mutation of the *hns* gene and transposition events, which may occur in dependence on the gene repertoire of specific strains. Thus papillae formation is a strong indicator for the presence of a functional but silent (cryptic) *bgl* operon. To analyze whether the *bgl* operon is silent but functionally intact or whether it is mutated, the Bgl phenotype of all strains of the collection was tested on salicin indicator plates. In this analysis three phenotypes could be distinguished, two of which were as expected. Firstly, the formation of Bgl-positive papillae within Bgl-negative colonies was observed indicating that the *bgl* operon is functional but silent. Secondly, a Bgl-negative phenotype was observed for strains which do not carry the *bgl* operon and for strains which presumably carry a mutated, non-functional *bgl* operon. The wide spectrum of mutations which relieve silencing of *bgl* should allow papillae formation in all strain backgrounds, as long as the operon is intact. All strains with deletions or insertions in the *bglGFB* genes (Figure S2) did not form Bgl-positive papillae. Interestingly, some strains showed a third phenotype. These strains were Bgl-negative on day one of incubation but turned weakly Bgl-positive after 2 to 4 days of incubation at 37°C (Figure 4A). To verify that this phenotype is not caused by mutations, the Bgl-positive colonies were re-streaked. Upon re-streaking the colonies again had a negative phenotype on day one and turned weakly positive after 2 to 4 days of incubation (Figure 4A). Quantification of this weak positive phenotype from colonies is difficult. However, the expression level was sufficiently high to prevent outgrowth of Bgl-positive papillae at 37°C. A similar result was obtained by Brooks et al. (1980, 1981) who analyzed uropathogenic *E. coli* [40],[41] (and see below). Intriguingly, one of the strains with a weak Bgl-positive phenotype at 37°C was the septicemic strain i484, for which expression of *bgl* was shown upon infection of mouse [34]. However, all strains with a weak Bgl-positive phenotype at 37°C were Bgl-negative when grown at 28°C. In addition, at 28°C Bgl-positive mutants appeared as papillae. Of strain i484 two Bgl-positive mutants which grew as papillae at 28°C were picked, re-streaked and analyzed. One mutant carried a 47 bp deletion of the H-NS binding region located upstream of the *bgl* promoter and CRP-binding site, and a second mutant carried a point mutation within the CRP-binding site identical to a mutation isolated before in *E. coli* K12 [22]. This suggests that the *bgl* operon in i484 is repressed by H-NS, and that repression by H-NS is relaxed at 37°C.

In order to associate the Bgl-phenotype with the clonal structure of the collection, the phenotypes were color-coded and mapped on the MS_TREE_ (Figure 4B). This visualization demonstrated a strong correlation of the functionality of *bgl* with the diversification of the *bgl* locus and with the clonal structure of the *E. coli* collection. Remarkably, in strains belonging to the ST73, ST95, and ST12 complexes and closely related STs (which all correspond to the phylogenetic group B2), the *bgl* operon was functional (i.e. structurally intact) in all but two strains, in contrast to strains belonging to other phylogenetic groups (see below). Furthermore, only in strains of these and related sequence types silencing of *bgl* was relaxed and the operon was weakly expressed in approximately 50% of the isolates. The prevalence of the weak Bgl-positive phenotype was highest in strains of the clonal groups ST73 and 12, which may suggest that the presence of this phenotype provides an advantage for these strains. In contrast, in strains of the ST10 and ST23 complexes as well as in other STs which correspond to the phylogenetic groups A, B1, and AxB1, the *bgl* operon was functional (papillation) in only 80% of the strains suggesting that 20% of type Ia or Ib *bgl* loci acquired mutations rendering the *bgl* operon non-functional (Figure 4B and Table S1). Further, in strains which carry a *bgl* type Ia locus integration of insertion elements in the *bglHIK*-*yieJI* genes is frequent (10 of 33 strains) (Figure S2, Table S1).

### Ancestry of the *bgl* Operon

**Figure 5:**
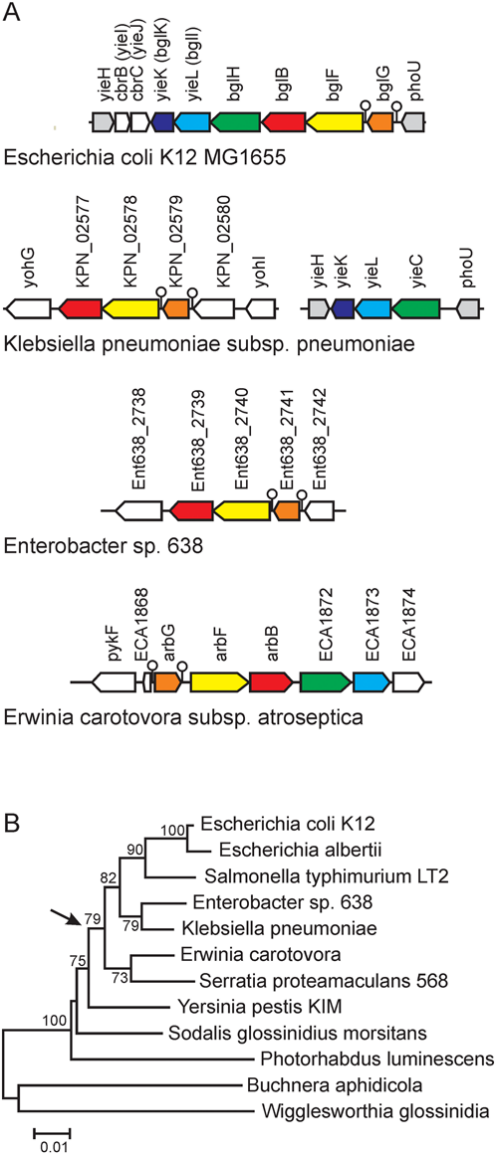
Orthologs of *bgl* in *Enterobacteriaceae*. (A) Gene arrangement of orthologs of the *bgl* operon present in *Klebsiella*, *Enterobacter*, and *Erwinia* and (B) NJ tree of 16S rDNA sequences of selected enterobacterial species. The arrow indicates the putative horizontal transfer of *bgl* from low GC-content Gram-positive bacteria. In (A) lollipop structures indicate conserved transcriptional terminators important for regulation by antitermination. The genes represented in the schemes are annotated with the following functions: *Klebsiella*: *yohI* (KPN_02581, Conserved protein, FMN-linked), KPN_02580 putative carbohydrate-selective porin (not homologous to *bglH*), KPN_02579 putative transcriptional antiterminator, KPN_02578 putative cellobiose-specific PTS permease, KPN_02577 putative phospho-β,D-glucosidase, KPN_02576 putative channel/filament proteins (*yohG*), as well as *yieC* Carbohydrate-specific outer membrane porin in cryptic operon (KPN_04128), *yieL* putative xylanase (KPN_04127), *yieK* putative uncharacterized protein (KPN_04126). For *Enterobacter*: Ent638_2738 RND efflux system, outer membrane lipoprotein, Ent638_2739 glycoside hydrolase, family 1, Ent638_2740 PTS system, β-glucoside-specific IIABC, Ent638_2741 transcriptional antiterminator, BglG, and Ent638_2742 dihydrouridine synthase, DuS. For *Erwinia* ECA1868 hypothetical protein, *arbG* β-glucoside operon antiterminator (ECA1869), *arbF* β-glucoside-specific PTS system components IIABC (ECA1870), *arbB* 6-phospho-β,D-glucosidase (ECA1871), ECA1872 putative porin, ECA1873 putative glycosyl hydrolase exoenzyme, and ECA1874 putative inosine-uridine preferring nucleoside hydrolase.

Our analysis demonstrated that the *bgl* and Z5211-5124 loci are clonally inherited within the modern group of *E. coli* strains, while neither the *bgl*-*yieIJ* nor the Z5211-Z5214 gene clusters are present in strains of the second *E. coli* population and in *E. albertii*. *Salmonella* sp. likewise does not contain these genes. To analyze the ancestry of the *bgl*-*yieIJ* and Z5211-Z5214 genes, the NCBI non-redundant database including the completed proteobacterial genome sequences, and the UniProt database [42] were searched for orthologs. As query the deduced protein sequences encoded by the *E. coli* K12 *bgl*-*yieIJ* locus and the *E. coli* 0157∶H7 EDL933 Z5211-5214 locus, respectively, were used. This search identified orthologs of the *bgl* operon genes *bglG*, *bglF*, and *bglB* in the enterobacterial species *Klebsiella*, *Enterobacter*, and *Erwinia* (Table S3). Orthologs of *bglF* and *bglB* were also found in the γ-proteobacterium *Photorhabdus luminescens* and a *bglB* ortholog was identified in *Vibrio harveyi* (Table S3). In addition, orthologs of likewise high similarity were found for *bglH* and *bglI* (*yieL*) in *Klebsiella* and *Erwinia* species, and for *bglK* (*yieK*) in *Klebsiella* (Table S3). The chromosomal context of these orthologous genes in *Klebsiella*, *Enterobacter*, and *Erwinia* species is shown in Figure 5. Interestingly, in all enteric bacteria in which the first three genes of the *bgl* operon (*bglGFB*) are present they form similar units including two terminator sequences for regulation by transcriptional antitermination (Figure 5). However, the presumptive promoter regions located upstream of the first terminator are not homologs (not shown). The *bglGFB* homologs map at different chromosomal locations in *E. coli*, *Klebsiella*, *Erwinia*, and *Enterobacter*, while the orthologs of genes *bglH*, *yieL*, and *yieK* map at the same chromosomal position in *E. coli* and *Klebsiella* (Figure 5). Homologs of the *bglG*, *bglF*, and *bglB* genes, as well as of *bglK* are also present in low GC-content Gram-positive bacteria (Firmicutes) (Table S3). For *bglG*, *bglF*, and *bglB* and their orthologs NJ trees were constructed for phylogenetic analysis (Figure S5). The phylogeny of the orthologs identified in the γ-proteobacteria was rather similar to the species phylogeny. However, a *bglG* homolog in *Photorhabdus luminescens* was less similar than homologs identified in Gram-positive bacteria, and in the BglF tree the ortholog present in *Erwinia carotovora* seems closely related to *E. coli* BglF (Figure 5). Taken together the data indicate that the *bglG*-*bglF*-*bglB* gene cluster was assembled in a common ancestor of *Erwinia*, *Klebsiella*, *Enterobacter*, and *E. coli*. The homologies to proteins of Gram-positives indicate that the genes were acquired by γ-proteobacteria by one or several horizontal transfer events from low-GC-content Gram-positives.

In contrast to the *bgl* operon genes, no orthologs for genes *yieI* (*cbrB*) and *yieJ* (*cbrC*) were found in enterobacterial genomes other than *E. coli* and *Shigella* sp. For YieJ, homologs of 40 to 50 percent identity are present in Firmicutes (Table S3). In contrast, a BLAST search for homologs of YieI yielded only weak hits in *Salmonella* (30% identity) (Table S3). Taken together the data suggest that the *yieI* and *yieJ* genes were acquired by horizontal transfer events, as proposed before [43]. For genes Z5211 to Z5214 no homologs were identified in the entire non-redundant NCBI-database in agreement with a previous analysis in which these genes were defined as ORFans [5]. It is interesting though that the proteins putatively encoded by Z5211 and Z5214 are 50% homologous to each other. Further, the GC-content of the Z5211-5214 cluster is only 30% and thus significantly lower than the average GC-content of the *E. coli* K12 genome (50.4%). Taken together, this suggests that the Z5211 to Z5214 genes were acquired horizontally.

## Discussion

The *bgl* operon of *E. coli* is a classical example of a locus which is repressed by H-NS, and is often referred to as being cryptic, since no laboratory conditions are known which induce its expression. Here we have shown that the evolution and functional state of the *bgl* operon is tightly coupled to the phylogeny of *E. coli*. The *bgl* operon is maintained functionally in strains of the phylogenetic group B2 of *E. coli*, and silencing of *bgl* by H-NS is less strict in roughly half of the strain belonging to this group. In contrast, the *bgl* operon is subject to erosion in the phylogenetic groups A and B1, and was presumably replaced by a cluster of ORFan genes (Z-locus) in strains belonging to the phylogenetic group D. Taken together these results indicate that the *bgl* operon provides a selective advantage in strains of the phylogenetic group B2, which includes uropathogenic and other extra-intestinal pathogens. Possibly, in strains of this group relief of silencing by transcriptional regulator proteins LeuO and BglJ may occur under certain conditions *in vivo*. Erosion of *bgl* in commensal *E. coli* and intestinal pathogens belonging to the phylogenetic groups A and B1 and loss of *bgl* in D strains may suggest that the locus is evolving neutrally or that it may even provide a selective disadvantage under certain conditions. Additional phylogenetic analyses suggest that *bgl* operon genes were acquired by horizontal transfer from low GC-content Gram-positive bacteria to an ancestral Enterobacterium.

Sequence based typing of the *bgl* operon and comparison of the *bgl* operon sequences from 17 genomes revealed the existence of 3 types (Ia, Ib, and II) of the operon with characteristic differences in their sequence. Mapping of these *bgl* sequence types onto a minimal spanning tree of a representative collection of *E. coli* uncovered a strong correlation of the *bgl* types with the clonal structure and phylogeny of *E. coli*. Likewise, comparison of a NJ tree based on the *bgl* sequences extracted of 17 genomes with a phylogenetic tree based on 7 house keeping genes revealed a strong congruence. Among the 171 strains of the collection and among the 17 sequenced strains only two recombination events between strains of different phylogenetic groups became apparent.

Analysis of the functional state of the *bgl* operon in the representative collection of *E. coli* using a papillation assay revealed a further interesting correlation. The *bgl* operon was found to be non-functional in 20% of the A and B1 strains as well as in hybrid strains, which carry type Ia or Ib *bgl* loci. Strains which carry the Z-locus instead of *bgl* were Bgl-negative, as expected. However, in the phylogenetic group B2 *bgl* was functional in almost all strains, with only 2 mutants among 48 strains. Furthermore, at 37°C approximately 50% of the B2 strains revealed a weak Bgl-positive phenotype. At 28°C, these strains were Bgl-negative, and Bgl-positive papillae appeared. Two such Bgl-positive papillae isolated of strain i484 were characterized and found to carry a deletion of the H-NS binding site and a point mutation in the CRP-site, respectively. Both mutations are known to relieve H-NS mediated repression in *E. coli* K12 [20]. Taken together these data suggest that silencing by H-NS is less strict (‘relaxed’) in B2 strains at 37°C. Interestingly, this relaxed phenotype is most prevalent in B2 strains belonging to the ST73 complex and to STs 127 and 12. Most MLST typed UPEC strains also belong to these STs (as listed in the E. coli MLST database). A similar correlation of uropathogenicity with the ability to ferment aryl-β,D-glucosides was reported before [40],[41]. Silencing of the *bgl* operon is also relaxed in some septicemic and other isolates, including the septicemic strain i484, for which expression of *bgl* was detected upon infection of mouse liver [34]. The molecular mechanism underlying the relaxed phenotype is unlikely to be caused by single nucleotide polymorphisms between the type II *bgl* operon present in B2 strains and the type Ia and Ib *bgl* loci prevalent in A and B1 strains. The *bgl* operon from strain i484 (and other strains in which *bgl* is weakly expressed) is silent in the laboratory strain K12, as tested using *lacZ* fusions to the *bgl* promoter and regulatory region (data not shown). Thus, the relaxed phenotype is presumably strain-specific. However, presently the molecular basis of the relaxed phenotype remains unclear. A genetic screen for mutants of i484 in which *bgl* is silent at 37°C yielded mutations in genes having pleiotropic effects (not shown). Genome comparisons are unlikely to allow the identification of loci, which may be important for the relaxed phenotype, since the gene repertoire of strains belonging to the same phylogenetic groups differ by more than 15% from strain to strain (data not shown) [5].

The *bgl* operon is often referred to as cryptic, as for *E. coli* K12 no laboratory growth conditions are known at which expression of *bgl* can be induced. However, the transcriptional regulators LeuO and BglJ counteract repression of *bgl* by H-NS [24],[44],[45]. Relief of repression by LeuO and BglJ requires constitutive expression of *leuO* and *bglJ*, respectively, since both genes are likewise repressed by H-NS at laboratory growth conditions [46],[47]. However, LeuO is a known pathogen determinant in *Salmonella enterica* [48]–[50], while BglJ is co-encoded in an operon with YjjQ, which is presumably important for infections by avian pathogenic *E. coli* [51]. Taken together, it is conceivable that silencing of *bgl* can be relieved under certain conditions *in vivo*, in agreement with the finding that *bgl* becomes induced in the septicemic strain i484 upon infection of mouse liver [34]. Considering that *bgl* is conserved in extra-intestinal pathogenic *E. coli*, conditions encountered in the extra-intestinal environment may be crucial. Indeed it was found that expression of the *bgl* operon can provide a selective advantage at certain conditions. This was analyzed in *E. coli* K12 were the presence of a mutationally ‘activated’ *bgl* operon provided a selective advantage in stationary phase in an *rpoS* mutant, although not in the K12 wild-type [52]. Furthermore, expression of the wild-type *bgl* operon in K12 occurs at a low level in stationary phase and is reduced in a *bglJ* mutant [53].

The laboratory strain K12 belongs to the phylogenetic group A of *E. coli*. In K12 the *bgl* operon is functional and can be ‘activated’ by mutations which relieve repression by H-NS. This phenomenon was discovered long before a general awareness of *in vivo* induction of genes in the host environment and of bacterial genome variation was developed. Therefore, it was assumed that mutational activation is required for expression of *bgl*, and it was speculated that mutational activation may provide a selective advantage for the population. However, the present study does not add to this model. The *bgl* operon can be activated by mutations under laboratory growth conditions in all *E. coli* strains in which it is present in a functional state, as evident by papillae formation on salicin plates. Activation of *bgl* occurs in strains belonging to the phylogenetic groups A, B1 and B2. However, the operon is conserved in a functional state in B2 strains only, while it is mutated in 20% of the other strains. This suggests that activation of *bgl* by mutations and a concomitant rare phenotypic variation does not provide a fitness advantage sufficiently high to positively select for a functional operon in A and B1 strains as well as in AxB1and ABD recombinants. The *bgl* operon is inactivated by mutations in all *Shigella* strains (which belong to the *E. coli* species). Further, in all strains of the phylogenetic group D (which includes many intestinal pathogens) the *bgl* operon was presumably replaced by a cluster of ORFan genes. Similarly, early analyses of the prevalence and functional state of *bgl* in *E. coli* demonstrated that the operon is present in many strains and silent in almost all of them. It was also detected that the *bgl* operon was inactive or lost and replaced by another fragment of DNA in some strains [15]. Erosion of the *bgl* operon may indicate that it does not provide a significant fitness advantage in the intestinal habitat, while the accumulation of *bgl* mutants or the replacement of *bgl* in enteroinvasive (EIEC) and enterohemorrhagic *E. coli* (EHEC) may be indicative of a fitness disadvantage of *bgl* in these pathogenic *E. coli*.

The evolution of the *bgl* locus strikingly correlates with the clonal structure and phylogeny of *E. coli*. Interestingly, *bgl* is absent in the closely related species *E. albertii* and it is also absent in the rare representatives of a second population of *E. coli* which presumably diverged early in evolution. However, orthologs of more than 70% similarity are present in the enterobacterial species *Klebsiella*, *Enterobacter*, and *Erwinia*. Less similar homologs were detected in *Bacillus* and *Listeria* and other low GC-content Gram positives. This suggests that the *bgl* operon genes were transferred to an ancestral enterobacterial species from low GC-content Gram-positives, then vertically inherited and retained in *Erwinia*, *Klebsiella*, and *Enterobacter*. One possibility is that *bgl* was vertically inherited to *E. coli* and in parallel lost in the closely related enterobacterial species *Salmonella* sp., *Escherichia albertii*, and also in representatives of the second *E. coli* population. Another possibility is that the *bgl* locus was lost before *Salmonella* and *Escherichia* diverged and that it was regained by *E. coli* by a second horizontal transfer event from an Enterobacterium. In this respect, it is interesting that the *bglGFB* genes and their homologs in *Klebsiella*, *Enterobacter*, and *Erwinia* are encoded as operons of similar structure although at different chromosomal locations. The homologous operons all contain the regulatory signals required for regulation by transcriptional antitermination by the operon encoded antiterminator (BglG in *E. coli*) which binds to the RNA and prevents termination at two terminators located in the leader and between the regulatory and the structural genes [54]. This mechanism of regulation of catabolic operons by antitermination is prevalent in low GC-content Gram positive bacteria [27], and thus further supports that *bgl* originates from a horizontal transfer event from low GC-content Gram-positives. However, the promoter region of the *E. coli bgl* operon is different from that of *Erwinia*, *Klebsiella*, and *Enterobacter*. The *arb* operon in *Erwinia* is not silent [55],[56]. Similarly, the *bgl* homologs are presumably not silent in *Klebsiella* and *Enterobacter* strains, which show a β-glucoside positive phenotype [57],[58].

The fate of the *bgl* operon in *E. coli* and in evolution of *Enterobacteriaceae* differs from other loci which were analyzed at the population level. For example, in evolution of type 1 fimbrial genes several independent horizontal transfer events are likely to have occurred [59]. Further, the *bgl-yieJI* and the Z5211-Z5214 loci lack any apparent mobilization functions and they are not inserted next to a tRNA gene in contrast to many genomic and pathogenicity islands [12]. However, silencing by H-NS is a feature which is common for loci acquired by horizontal transfer [17],[18]. Further, horizontal transfer of genes for metabolic functions probably occurred repeatedly, for which transfer of genes coding for the phosphoenolpyruvate-dependent phosphotransfer system during the evolution of γ-proteobacteria provides one example [60]. The route of gain, inheritance, erosion and loss of *bgl* by enterobacteria and within *E. coli* is in agreement with genome scale analyses of gene gain and loss in the evolution of γ-proteobacteria as well as of *E. coli* [5],[6]. Our analysis of a single locus of the variable gene pool at the population level in combination with phylogenetic reconstruction provides a snapshot of ongoing evolution of a locus which was presumably acquired horizontally and then evolved clonally and differentially in commensal and pathogenic *E. coli*.

## Materials and Methods

### *E. coli* Strains

The *E. coli* strain collection analyzed in this study includes the strains of the ECOR reference collection [35] (obtained from Dr. Whittam, Michigan State University, USA), the uropathogenic strains 536 and J96 (obtained from Dr. Dobrindt, Universität Würzburg, Germany), representatives of a second population of *E. coli* [8]; the septicemic strain i484 [34] (obtained from Dr. Isaacson, University of Illinois, USA), the *E. albertii* strains (provided by Dr. Wieler, FU Berlin, Germany), and *E. coli* strains isolated at the local Institute for Medical Microbiology, Immunology and Hygiene. The strains are listed in supplementary Table S1.

### Typing of the Strain Collection

Multi locus sequence typing (MLST) data for the strains of the ECOR collection, as well as the uropathogenic strains J96 and 536, were taken from the *E. coli* MLST database (http://www.ucc.ie/mlst). MLST of the remaining strains was performed, as described [8]. Briefly, PCR primers and PCR reaction conditions were used according to the protocol available at the *E. coli* MLST database (http://www.ucc.ie/mlst). For sequencing of *adk*, *fumC*, and *recA* additional internal primers were used (S776 and S777 for *adk*; S778 for *fumC*; S766 and S767 for *recA*; Table S3). DNA sequencing was performed using BigDye Terminator Cycle Sequencing Kit (v1.1 and v3.1, Applied Biosystems) and an automated DNA sequencer run by the Cologne Center for Genomics. Analysis of the sequences including the assignment of alleles, sequence types, and ST complexes was performed using Bionumerics software (Applied Maths, NV). Minimum spanning trees (MS_TREE_) were generated using the Bionumerics software (Applied Maths, NV), as described [8]. Likewise, phylogenetic analysis of the collection and assignment of phylogenetic groups to new strains was performed, as described [8]. Briefly, for phylogenetic analysis the sequences of the 7 MLST loci were concatenated and aligned using ClustalW, and phylogenetic trees were constructed using the neighbor-joining algorithm with default parameters and 1000 bootstrap replicates using the MEGA4 (http://www.megasoftware.net/) [61]. The assignment of the phylogenetic groups was performed using STRUCTURE, as described [8]. The sequences used for phylogenetic analyses are provided in the supplement (Dataset S1).

### Typing of *bgl* Operon and the Z5211-5214 Locus

Typing of the *bgl* operon and the Z5211-5214 locus was performed by PCR with a collection of primers (Table S4) matching to the flanking genes of the core genome and within the *bgl-yieIJ* and Z5211-Z5214 loci. For some strains the PCR typing revealed insertions or deletions within the *bgl-yieI-yieJ* and Z5211-Z5214 loci. These were further characterized by sequencing (Figure S2). For phylogenetic analysis of the *bgl-yieI-yieJ* and the Z5211-Z5214 islands, PCR fragments encompassing the fusions of the core genes *phoU* and *yieH*, respectively, to the island were sequenced on both strands. The sequences were manually curated using the ContigExpress program of Vector NTI Suite (Invitrogen) and Bionumerics software (Applied Maths NV). Subsequently, the sequences derived from the two flanking regions of the *bgl-yieI-yieJ* and Z5211-5214 islands, were trimmed to include only island specific sequences. These trimmed sequences were concatenated, aligned and analyzed by construction of NJ trees with the MEGA4 (http://www.megasoftware.net/) using default parameters and 1000 bootstrap replicates. The sequences obtained for *E. albertii* and the rare *E. coli* strains representing the second population were deposited at the NCBI Genbank database (accession numbers EU056311, EU056312, EU056313, and EU056314).

### Homology Searches

For identifying homologs of the genes encoded by the *bgl* operon and by *yieI* and *yieJ*, as well as for the putative genes of the Z5211-Z5214 locus, the deduced protein sequences from *E. coli* K12 MG1655 and O157∶H7 EDL933, respectively, were used as query to search the NCBI microbial genomes database as well as the NCBI non-redundant nucleotide database using TBLASTN. In parallel, the UniProt database [62] was searched for homologs using BLASTP. Similar results were obtained in these searches and the protein sequences of the homologs were downloaded for phylogenetic analysis, by alignment and construction of NJ trees. The sequences used are provided in the supplement (Dataset S1). For comparison, 16S rDNA sequences of enterobacterial representatives were obtained from the Integrated Microbial Genomes (IMG) database (http://img.jgi.doe.gov/cgi-bin/pub/main.cgi) [63], and used for construction of a species tree.

### Analysis of β-Glucoside Phenotypes

For analysis of the β-glucoside (Bgl) phenotype, the strains were streaked on Bromthymol Blue (BTB) salicin (β-glucoside) indicator plates [29]. The Bgl phenotypes and the appearance of Bgl-positive papillae, which are indicative of a functional but silent *bgl* operon, were documented up to 4 days of incubation at 37°C and 28°C, respectively.

## Supporting Information

Figure S1 Phylogeny of the strain collection represented with NJ trees. The NJ trees are based on the concatenated sequences of the MLST loci. The NJ tree shown to the right includes strains of the ECOR collection, while the tree shown to the left includes all other strains. For comparison ancestral *E. coli* and *E. albertii* strains are included in both trees. The phylogenetic groups are indicated next to the designations of the strains. Numbers on nodes are bootstrap scores from 1000 replicates.
(0.18 MB TIF)

Figure S2 Insertion and deletion mutations (indels) in the *bgl-yieIJ* and Z5211-Z5214 loci. Strains which carry insertion mutations and/or deletions were characterized by sequencing of the insertion and/or deletions sites. The mutants are grouped by *bgl* locus types Ia, Ib, and II, as well as the Z-locus type III. The definition of these types is based on structural and sequence analyses (Figs. 1, 2, and S4). The strain names are indicated to the left, the mutations are schematically shown and the Bgl phenotypes of these strains are indicated to the right. P indicates presence of a silent *bgl* operon, which can be activated by spontaneous mutations appearing as papillae on indicator plates, ‘+’ indicates weakly Bgl-positive, and ‘−’ indicates a Bgl-negative phenotype (even after prolonged incubation). The number of strains (No.) which carry *bgl-yieIJ* (or Z5211-Z5214 loci) of similar structure and which have identical Bgl phenotypes is given to the right of the schemes.
(0.17 MB TIF)

Figure S3 Southern analysis of strains which do not carry the *bgl* operon. Genomic DNA of the strains indicated was digested with EcoRI and EcoO109. The DNA was separated by agarose gel electrophoresis and blotted onto a Nylon membrane. The membrane was hybridized with a radioactive probe encompassing the *bglG-bglF* region (shown here) as well as with other probes specific for other regions of the bgl-yieIJ locus (not shown). Genomic DNA of strains MG1655 and i484 were used as positive controls. The Southern analyses, which were performed for the strains indicated in table S1, confirmed that these strains do not carry genes of the *bgl-yieIJ* locus elsewhere in the genome.
(0.31 MB TIF)

Figure S4 Phylogeny of the *bgl-yieI* locus. The sequences of the left and right end of the *bgl*-*yieI* island were concatenated, aligned and used for construction of NJ trees. The NJ tree shown to the right includes strains of the ECOR collection, while the tree shown to the left includes all other strains. For comparison in both trees the respective sequences from *E. coli* and *Shigella flexneri* genomes were included. The NJ trees show 3 well separated clades, which provide the base to define types Ia, Ib, and II to the *bgl* locus. Numbers on nodes are bootstrap scores from 1000 replicates.
(0.62 MB TIF)

Figure S5 Phylogenetic analysis of BglG, BglF, and BglB homologs. The sequences of homologs were aligned with CLUSTALW and NJ trees were constructed with MEGA4. For every genus one representative was chosen (listed in Table S3).
(0.31 MB TIF)

Table S1 *E. coli* and *E. albertii* strains.
(0.37 MB DOC)

Table S2 Genome sequences used.
(0.05 MB DOC)

Table S3 Homologs of the E. coli *bgl-yieIJ* locus.
(0.08 MB DOC)

Table S4 Oligonucleotides used for MLST and *bgl*-Z typing.
(0.12 MB DOC)

Dataset S1 Sequences used for phylogenetic analyses.
(0.11 MB ZIP)

## References

[1] Blattner FR, Plunkett G, Bloch CA, Perna NT, Burland V (1997). The complete genome sequence of *Escherichia coli* K-12.. Science.

[2] Perna NT, Plunkett G, Burland V, Mau B, Glasner JD (2001). Genome sequence of enterohaemorrhagic *Escherichia coli* O157∶H7.. Nature.

[3] Hayashi T, Makino K, Ohnishi M, Kurokawa K, Ishii K (2001). Complete genome sequence of enterohemorrhagic *Escherichia coli* O157∶H7 and genomic comparison with a laboratory strain K-12.. DNA Res.

[4] Welch RA, Burland V, Plunkett G, Redford P, Roesch P (2002). Extensive mosaic structure revealed by the complete genome sequence of uropathogenic *Escherichia coli*.. Proc Nat Acad Sci.

[5] van Passel MW, Marri PR, Ochman H (2008). The Emergence and Fate of Horizontally Acquired Genes in Escherichia coli.. PLoS Comput Biol.

[6] Lerat E, Daubin V, Ochman H, Moran NA (2005). Evolutionary origins of genomic repertoires in bacteria.. PLoS Biol.

[7] Herzer PJ, Inouye S, Inouye M, Whittam TS (1990). Phylogenetic distribution of branched RNA-linked multicopy single-stranded DNA among natural isolates of *Escherichia coli*.. J Bacteriol.

[8] Wirth T, Falush D, Lan R, Colles F, Mensa P (2006). Sex and virulence in *Escherichia coli*: an evolutionary perspective.. Mol Microbiol.

[9] Maiden MCJ (2006). Multi locus Sequence Typing of Bacteria.. Annu Rev Microbiol.

[10] Escobar-Paramo P, Giudicelli C, Parsot C, Denamur E (2003). The Evolutionary History of *Shigella* and Enteroinvasive *Escherichia coli*.. J Mol Evol.

[11] Achtman M, Wagner M (2008). Microbial diversity and the genetic nature of microbial species.. Nat Rev Micro.

[12] Dobrindt U, Hochhut B, Hentschel U, Hacker J (2004). Genomic islands in pathogenic and environmental microorganisms.. Nat Rev Microbiol.

[13] Schaefler S, Maas WK (1967). Inducible System for the Utilization of β-Glucosides in *Escherichia coli* II. Description of mutant types and genetic analysis.. J Bacteriol.

[14] Reynolds AE, Felton J, Wright A (1981). Insertion of DNA activates the cryptic *bgl* operon of *E. coli* K12.. Nature.

[15] Hall BG (1988). Widespread distribution of deletions of the *bgl* operon in natural isolates of *Escherichia coli*.. Mol Biol Evol.

[16] Hall BG, Yokoyama S, Calhoun DH (1983). Role of cryptic genes in microbial evolution.. Mol Biol Evol.

[17] Dorman CJ (2007). H-NS, the genome sentinel.. Nat Rev Micro.

[18] Navarre WW, McClelland M, Libby SJ, Fang FC (2007). Silencing of xenogeneic DNA by H-NS-facilitation of lateral gene transfer in bacteria by a defense system that recognizes foreign DNA.. Genes Dev.

[19] Schaefler S (1967). Inducible System for the Utilization of b-Glucosides in *Escherichia coli*. I. Active Transport and Utilization of β-Glucosides.. J Bacteriol.

[20] Schnetz K (1995). Silencing of *Escherichia coli bgl* promoter by flanking sequence elements.. EMBO J.

[21] Mukerji M, Mahadevan S (1997). Characterization of the negative elements involved in silencing the *bgl* operon of *Escherichia coli*: possible roles for DNA gyrase, H-NS, and CRP-cAMP in regulation.. Mol Microbiol.

[22] Reynolds AE, Mahadevan S, LeGrice SFJ, Wright A (1986). Enhancement of bacterial gene expression by insertion elements or by mutation in a CAP-cAMP binding site.. J Mol Biol.

[23] Dole S, Nagarajavel V, Schnetz K (2004). The histone-like nucleoid structuring protein H-NS represses the *Escherichia coli bgl* operon downstream of the promoter.. Mol Microbiol.

[24] Madhusudan S, Paukner A, Klingen Y, Schnetz K (2005). Independent regulation of H-NS mediated silencing of the *bgl* operon at two levels: upstream by BglJ and LeuO and downstream by DnaKJ.. Microbiology.

[25] Nagarajavel V, Madhusudan S, Dole S, Rahmouni AR, Schnetz K (2007). Repression by binding of H-NS within the transcription unit.. J Biol Chem.

[26] Stülke J, Arnaud M, Rapoport G, Martin-Verstraete I (1998). PRD - a protein domain involved in PTS-dependent induction and carbon catabolite repression of catabolic operons in bacteria.. Mol Microbiol.

[27] Görke B, Stülke J (2008). Carbon catabolite repression in bacteria: many ways to make the most out of nutrients.. Nat Rev Micro.

[28] Reichenbach B, Breustedt DA, Stülke J, Rak B, Görke B (2007). Genetic Dissection of Specificity Determinants in the Interaction of HPr with Enzymes II of the Bacterial Phosphoenolpyruvate:Sugar Phosphotransferase System in Escherichia coli.. J Bacteriol.

[29] Schnetz K, Toloczyki C, Rak B (1987). β-Glucoside (*bgl*) operon of *Escherichia coli* K-12: nucleotide sequence, genetic organization, and possible evolutionary relationship to regulatory components of two *Bacillus subtilis* genes.. J Bacteriol.

[30] Navarre WW, Porwollik S, Wang Y, McClelland M, Rosen H, Libby SJ (2006). Selective silencing of foreign DNA with low GC content by the H-NS protein in *Salmonella*.. Science.

[31] Lucchini S, Rowley G, Goldberg MD, Hurd D, Harrison M (2006). H-NS Mediates the Silencing of Laterally Acquired Genes in Bacteria.. PLoS Pathogens.

[32] Oshima T, Ishikawa S, Kurokawa K, Aiba H, Ogasawara N (2006). *Escherichia coli* histone-like protein H-NS preferentially binds to horizontally acquired DNA in association with RNA Polymerase.. DNA Res.

[33] Dorman CJ (2004). H-NS: a universal regulator for a dynamic genome.. Nat Rev Microbiol.

[34] Khan MA, Isaacson RE (1998). In vivo expression of the β-glucoside (*bgl*) operon of *Escherichia coli* occurs in mouse liver.. J Bacteriol.

[35] Ochman H, Selander RK (1984). Standard reference strains of *Escherichia coli* from natural populations.. J Bacteriol.

[36] Albert MJ, Alam K, Islam M, Montanaro J, Rahaman AS (1991). *Hafnia alvei*, a probable cause of diarrhea in humans.. Infect Immun.

[37] Brzuszkiewicz E, Bruggemann H, Liesegang H, Emmerth M, Olschlager T (2006). How to become a uropathogen: Comparative genomic analysis of extraintestinal pathogenic Escherichia coli strains.. Proc Nat Acad Sci.

[38] Escobar-Paramo P, Sabbagh A, Darlu P, Pradillon O, Vaury C (2004). Decreasing the effects of horizontal gene transfer on bacterial phylogeny: the Escherichia coli case study.. Mol Phylogenet Evol.

[39] Neelakanta G (2005). Genome variations in commensal and pathogenic *E. coli*.

[40] Brooks HJ, Benseman BA, Peck J, Bettelheim KA (1981). Correlation between uropathogenic properties of *Escherichia coli* from urinary tract infections and the antibody-coated bacteria test and comparison with faecal strains.. J Hyg (Lond).

[41] Brooks HJ, O'Grady F, McSherry MA, Cattell WR (1980). Uropathogenic properties of Escherichia coli in recurrent urinary-tract infection.. J Med Microbiol.

[42] Liu Z, Mukherjee A, Lutkenhaus J (1999). Recruitment of ZipA to the division site by interaction with FtsZ.. Mol Microbiol.

[43] Lawrence JG, Ochman H (1998). Molecular archaeology of the Escherichia coli genome.. Proc Natl Acad Sci.

[44] Ueguchi C, Ohta T, Seto C, Suzuki T, Mizuno T (1998). The *leuO* gene-product has a latent ability to relieve the *bgl* silencing in *Escherichia coli*.. J Bacteriol.

[45] Giel M, Desnoyer M, Lopilato J (1996). A mutation in a new gene, *bglJ*, activates the *bgl* operon in *Escherichia coli* K-12.. Genetics.

[46] Stratmann T, Madhusudan S, Schnetz K (2008). Regulation of the *yjjQ*-*bglJ* operon, encoding LuxR-type transcription factors, and the divergent *yjjP* gene by H-NS and LeuO.. J Bacteriol.

[47] Chen CC, Chou MY, Huang CH, Majumder A, Wu HY (2005). A cis-spreading nucleoprotein filament is responsible for the gene silencing activity found in the promoter relay mechanism.. J Biol Chem.

[48] Hernandez-Lucas I, Gallego-Hernandez AL, Encarnacion S, Fernandez-Mora M, Martinez-Batallar AG (2008). The LysR-Type Transcriptional Regulator LeuO Controls Expression of Several Genes in Salmonella enterica Serovar Typhi.. J Bacteriol.

[49] Rodriguez-Morales O, Fernandez-Mora M, Hernandez-Lucas I, Vazquez A, Puente JL (2006). *Salmonella enterica* serovar Typhimurium *ompS1* and *ompS2* mutants are attenuated for virulence in mice.. Infect Immun.

[50] Tenor JL, McCormick BA, Ausubel FM, Aballay A (2004). *Caenorhabditis elegans*-based screen identifies *Salmonella* virulence factors required for conserved host-pathogen interactions.. Curr Biol.

[51] Li G, Ewers C, Laturnus C, Diehl I, Dai J, Antão E-M (2008). Characterization of a *yjjQ* mutant of avian pathogenic *E. coli* (APEC).. Microbiology.

[52] Madan R, Kolter R, Mahadevan S (2005). Mutations that activate the silent *bgl* operon of *Escherichia coli* confer a growth advantage in stationary phase.. J Bacteriol.

[53] Madan R, Moorthy S, Mahadevan S (2008). Enhanced expression of the *bgl* operon of *Escherichia coli* in the stationary phase.. FEMS Microbiol Lett.

[54] Görke B (2003). Regulation of the Escherichia coli Antiterminator Protein BglG by Phosphorylation at Multiple Sites and Evidence for Transfer of Phosphoryl Groups between Monomers.. J Biol Chem.

[55] El Hassouni M, Henrissat B, Chippaux M, Barras F (1992). Nucleotide sequences of the *arb* genes, which control b- glucoside utilization in *Erwinia chrysanthemi*: Comparison with the *Escherichia coli bgl* operon and evidence for a new b-glycohydrolase family including enzymes from eubacteria, archebacteria, and humans.. J Bacteriol.

[56] El Hassouni M, Chippaux M, Barras F (1990). Analysis of the *Erwinia chrysanthemi arb* genes, which mediate metabolism of aromatic β-glucosides.. J Bacteriol.

[57] Raghunand TR, Mahadevan S (2003). The β-glucoside genes of Klebsiella aerogenes: conservation and divergence in relation to the cryptic *bgl* genes of *Escherichia coli*.. FEMS Microbiol Lett.

[58] Edberg SC, Pittman S, Singer JM (1977). Esculin hydrolysis by Enterobacteriaceae.. J Clin Microbiol.

[59] Weissman SJ, Chattopadhyay S, Aprikian P, Obata-Yasuoka M, Yarova-Yarovaya Y (2006). Clonal analysis reveals high rate of structural mutations in fimbrial adhesins of extraintestinal pathogenic Escherichia coli.. Mol Microbiol.

[60] Comas I, Gonzalez-Candelas F, Zuniga M (2008). Unraveling the evolutionary history of the phosphoryl-transfer chain of the phosphoenolpyruvate:phosphotransferase system through phylogenetic analyses and genome context.. BMC Evol Biol.

[61] Germon P, Roche D, Melo S, Mignon-Grasteau S, Dobrindt U (2007). tDNA locus polymorphism and ecto-chromosomal DNA insertion hot-spots are related to the phylogenetic group of Escherichia coli strains.. Microbiology.

[62] The UniProt Consortium (2008). The Universal Protein Resource (UniProt).. Nucleic Acids Res.

[63] Markowitz VM, Szeto E, Palaniappan K, Grechkin Y, Chu K (2007). The integrated microbial genomes (IMG) system in 2007: data content and analysis tool extensions.. Nucleic Acids Res.

[64] Prasad I, Schaefler S (1974). Regulation of the β-glucoside system in *Escherichia coli* K12.. J Bacteriol.

[65] Mahadevan S, Reynolds AE, Wright A (1987). Positive and negative regulation of the *bgl* operon in *Escherichia coli*.. J Bacteriol.

[66] Andersen C, Rak B, Benz R (1999). The gene *bglH* present in the *bgl* operon of *Escherichia coli*, responsible for uptake and fermentation of β-glucosides encodes for a carbohydrate-specific outer membrane porin.. Molecular Microbiol.

[67] de Vienne DM, Giraud T, Martin OC (2007). A congruence index for testing topological similarity between trees.. Bioinformatics.

