# Supplementary figures and images for: Fate of the H-NS–Repressed bgl Operon in Evolution of Escherichia coli
Source: PLoS Genet. 2009 Mar 6;5(3):e1000405. doi: 10.1371/journal.pgen.1000405 (PMC2646131; doi:10.1371/journal.pgen.1000405)

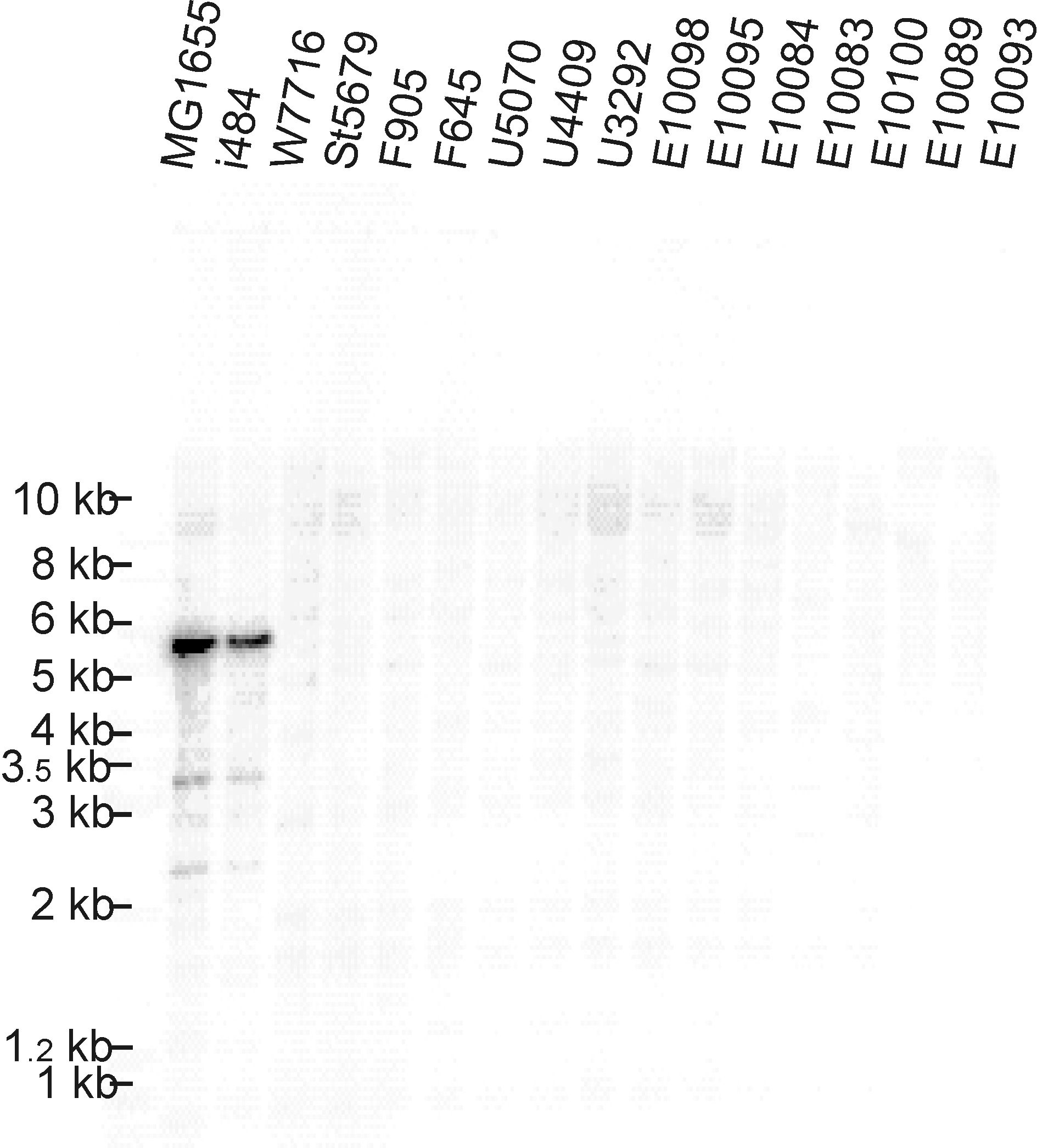

Supplement: Figure S3 — Southern analysis of strains which do not carry the bgl operon. Genomic DNA of the strains indicated was digested with EcoRI and EcoO109. The DNA was separated by agarose gel electrophoresis and blotted onto a Nylon membrane. The membrane was hybridized with a radioactive probe encompassing the bglG-bglF region (shown here) as well as with other probes specific for other regions of the bgl-yieIJ locus (not shown). Genomic DNA of strains MG1655 and i484 were used as positive controls. The Southern analyses, which were performed for the strains indicated in table S1, confirmed that these strains do not carry genes of the bgl-yieIJ locus elsewhere in the genome. (0.31 MB TIF) [file pgen.1000405.s003.tif]

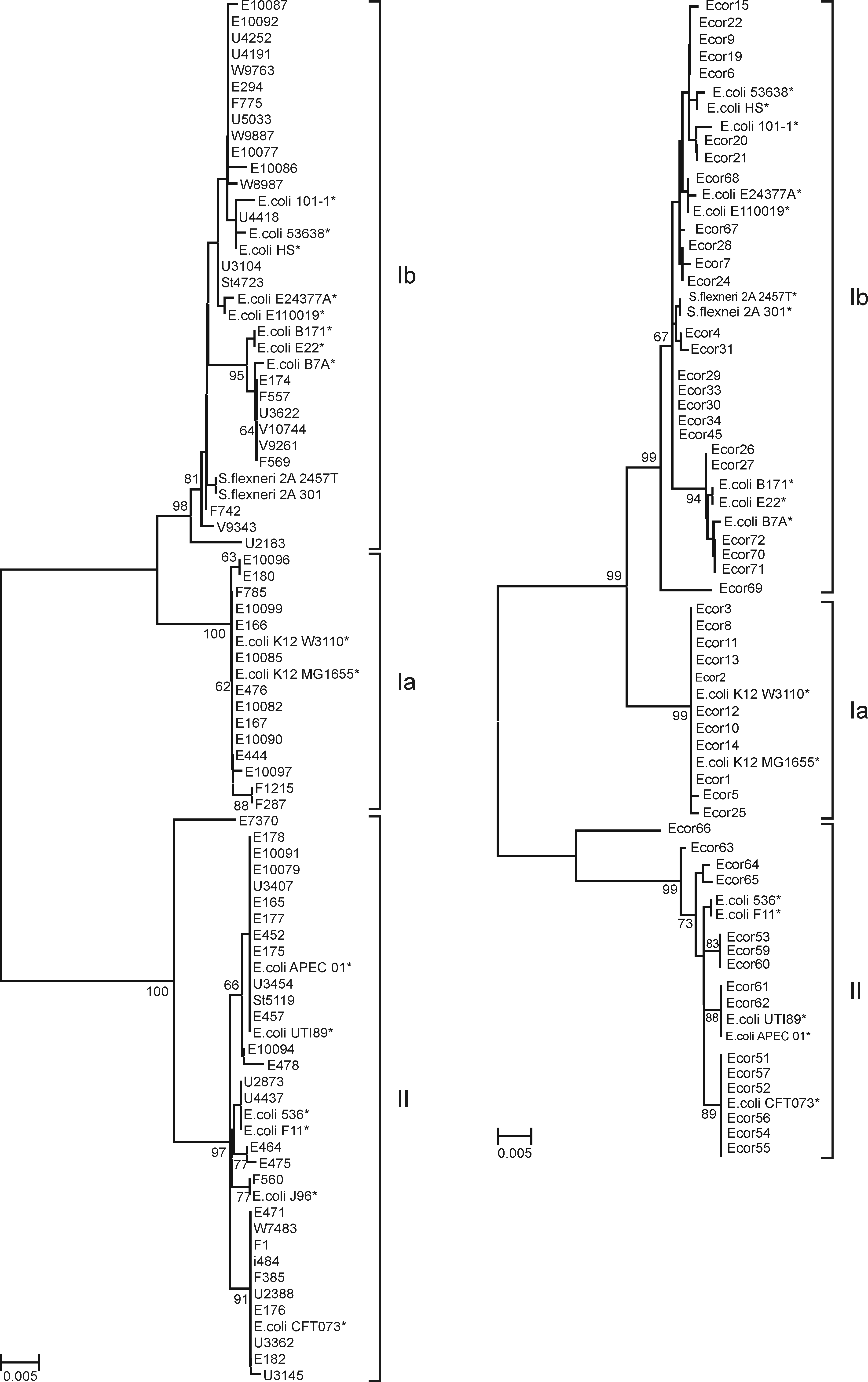

Supplement: Figure S4 — Phylogeny of the bgl-yieI locus. The sequences of the left and right end of the bgl-yieI island were concatenated, aligned and used for construction of NJ trees. The NJ tree shown to the right includes strains of the ECOR collection, while the tree shown to the left includes all other strains. For comparison in both trees the respective sequences from E. coli and Shigella flexneri genomes were included. The NJ trees show 3 well separated clades, which provide the base to define types Ia, Ib, and II to the bgl locus. Numbers on nodes are bootstrap scores from 1000 replicates. (0.62 MB TIF) [file pgen.1000405.s004.tif]

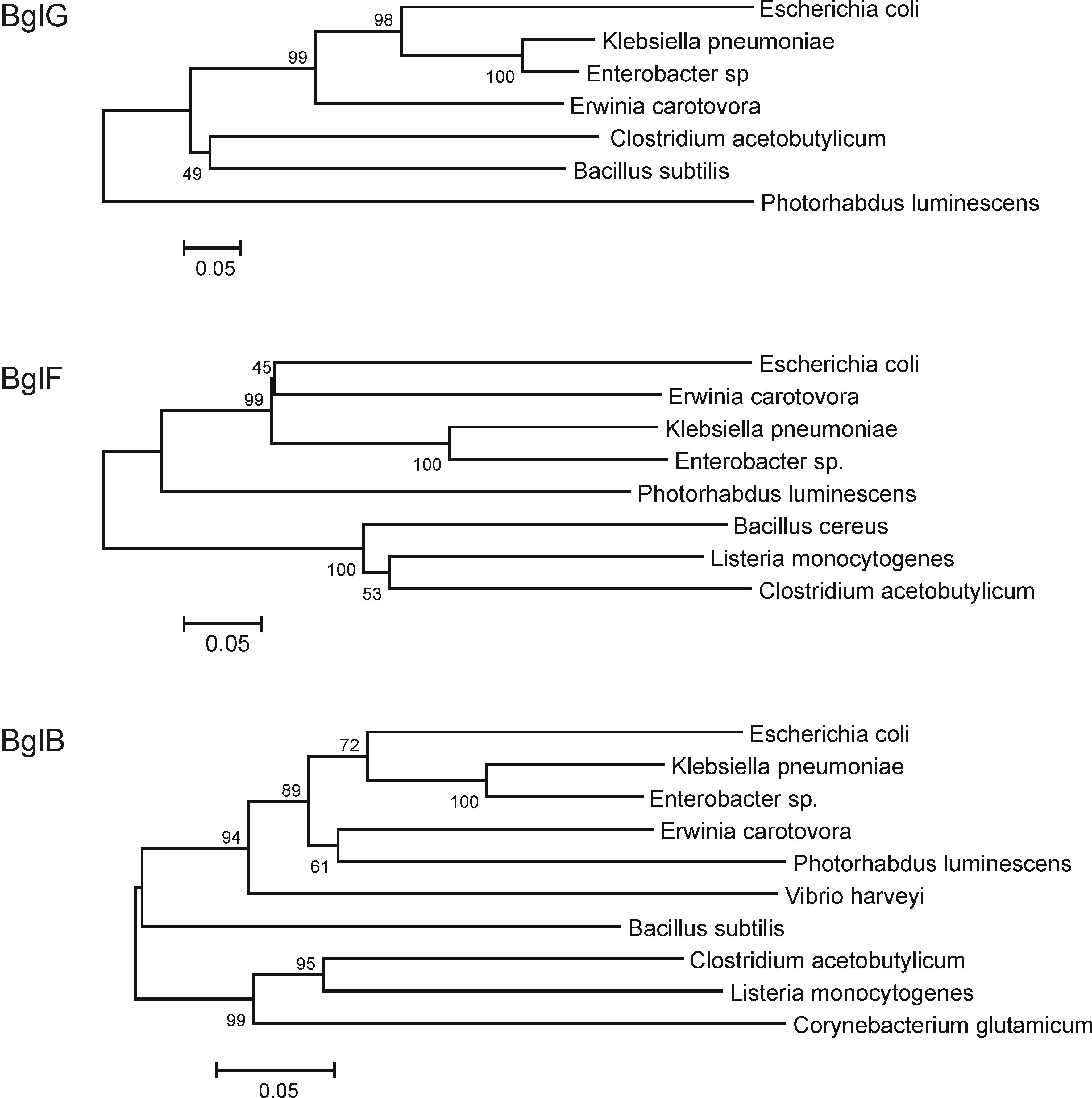

Supplement: Figure S5 — Phylogenetic analysis of BglG, BglF, and BglB homologs. The sequences of homologs were aligned with CLUSTALW and NJ trees were constructed with MEGA4. For every genus one representative was chosen (listed in Table S3). (0.31 MB TIF) [file pgen.1000405.s005.tif]
