# Supplementary material for: Fate of the H-NS–Repressed bgl Operon in Evolution of Escherichia coli
Source: PLoS Genet. 2009 Mar 6;5(3):e1000405. doi: 10.1371/journal.pgen.1000405 (PMC2646131; doi:10.1371/journal.pgen.1000405)
Supplement: Table S1 — E. coli and E. albertii strains. (0.37 MB DOC) [file pgen.1000405.s006.doc]

| **Table S1: *E. coli* and *E. albertii* strains** | | | | | | |
| --- | --- | --- | --- | --- | --- | --- |
| **Strain** | **ST^a^** | **ST complex** | **Phylogenetic group** | **bgl/Z type^b^** | **pheno­type^c^** | **Indels^d^** |
| E10082 | 10 | ST10 clx | A | Ia | 1 |  |
| E10085 | 10 | ST10 clx | A | Ia | 1 |  |
| E10090 | 10 | ST10 clx | A | Ia | 0 |  |
| E10097 | 10 | ST10 clx | A | Ia | 1 |  |
| E164 | 10 | ST10 clx | A | Ia | 1 | Δ(bglI-yieH)::IS1 |
| E166 | 10 | ST10 clx | A | Ia | 1 |  |
| E167 | 10 | ST10 clx | A | Ia | 1 | bglH::IS2 |
| E180 | 10 | ST10 clx | A | Ia | 1 |  |
| E291 | 10 | ST10 clx | A | Ia | 0 | yieJ::IS629 |
| E292 | 10 | ST10 clx | A | Ia | 1 | Δ(yieJ-yieI)::IS629 |
| E345 | 10 | ST10 clx | A | Ia | 1 | Δ(bglI-bglK)::IS629; yieJ::IS629 |
| E476 | 10 | ST10 clx | A | Ia | 1 |  |
| Ecor1 | 10 | ST10 clx | A | Ia | 0 |  |
| Ecor11 | 10 | ST10 clx | A | Ia | 1 |  |
| Ecor12 | 10 | ST10 clx | A | Ia | 1 | bglH::IS2 |
| Ecor14 | 10 | ST10 clx | A | Ia | 0 |  |
| Ecor25 | 10 | ST10 clx | A | Ia | 1 |  |
| Ecor3 | 10 | ST10 clx | A | Ia | 0 |  |
| Ecor5 | 10 | ST10 clx | A | Ia | 1 |  |
| Ecor8 | 10 | ST10 clx | A | Ia | 0 |  |
| F1215 | 10 | ST10 clx | A | Ia | 1 |  |
| F287 | 10 | ST10 clx | A | Ia | 1 |  |
| F785 | 10 | ST10 clx | A | Ia | 1 |  |
| U2366 | 10 | ST10 clx | A | Ia | 0 | bglB::IS186 |
| U3633 | 10 | ST10 clx | A | Ia | 1 | yieJ::ISEC8 |
| U4418 | 10 | ST10 clx | A | Ia | 1 | yieJ::IS1, yieJ::ISEC8 |
| U5107 | 10 | ST10 clx | A | Ia | 1 | Δ(bglK-yieJ)::IS629 |
| E10099 | 34 | ST10 clx | A | Ia | 1 |  |
| Ecor10 | 43 | ST10 clx | A | Ia | 1 |  |
| Ecor13 | 44 | ST10 clx | A | Ia | 1 |  |
| Ecor2 | 49 | ST10 clx | A | Ia | 1 |  |
| E10096 | 167 | ST10 clx | A | Ia | 1 | yieJ::ISEC8 |
| E444 | 548 | ST10 clx | A | Ia | 1 |  |
| Ecor18 | 48 | ST10 clx | A | ND | 0 | Δ(*bglR*-*bglF*)::IS1 |
| Ecor9 | 10 | ST10 clx | A | Ib | 0 | Δ(bglB-bglH)::IS1 |
| F557 | 23 | ST23 clx | B1 | Ib | 1 |  |
| Ecor15 | 45 | - | AxB1 | Ib | 1 |  |
| Ecor16 | 46 | ST46 clx | A | Ib | 0 |  |
| W9763 | 46 | ST46 clx | A | Ib | 1 |  |
| Ecor17 | 47 | - | AxB1 | Ib | 0 | Δ(*bgl*t1-*bglK*)::IS1 |
| Ecor19 | 48 | ST10 clx | A | Ib | 1 |  |
| Ecor20 | 48 | ST10 clx | A | Ib | 0 | bglB::IS1 |
| Ecor21 | 48 | ST10 clx | A | Ib | 0 | bglB::IS1 |
| U4252 | 48 | ST10 clx | A | Ib | 1 |  |
| W9887 | 48 | ST10 clx | A | Ib | 1 | bglH::IS1 |
| Ecor22 | 50 | - | A | Ib | 1 |  |
| Ecor24 | 52 | - | AxB1 | Ib | 0 |  |
| Ecor26 | 53 | - | AxB1 | Ib | 1 |  |
| Ecor27 | 53 | - | AxB1 | Ib | 1 |  |
| Ecor28 | 54 | - | AxB1 | Ib | 1 |  |
| Ecor29 | 55 | ST155 clx | B1 | Ib | 0 |  |
| Ecor30 | 56 | ST155 clx | B1 | Ib | 1 |  |
| Ecor33 | 56 | ST155 clx | B1 | Ib | 1 |  |
| Ecor31 | 57 | ST350 clx | AxB1 | Ib | 1 | ΔyieJ |
| Ecor34 | 58 | ST155 clx | AxB1 | Ib | 1 |  |
| Ecor4 | 63 | - | A | Ib | 1 | ΔyieJ |
| Ecor45 | 67 | - | AxB1 | Ib | 1 |  |
| Ecor58 | 75 | - | AxB1 | Ib | 1 | ΔyieJ |
| Ecor6 | 77 | ST206 clx | AxB1 | Ib | 0 |  |
| Ecor67 | 84 | - | AxB1 | Ib | 1 |  |
| Ecor68 | 85 | - | B1 | Ib | 1 |  |
| Ecor69 | 86 | ST86 clx | AxB1 | Ib | 1 | ΔyieJ |
| Ecor7 | 87 | - | AxB1 | Ib | 1 |  |
| E467 | 88 | ST23 clx | B1 | Ib | 1 |  |
| Ecor70 | 88 | ST23 clx | B1 | Ib | 1 |  |
| Ecor71 | 88 | ST23 clx | B1 | Ib | 1 |  |
| F569 | 88 | ST23 clx | B1 | Ib | 0 |  |
| U3622 | 88 | ST23 clx | B1 | Ib | 1 |  |
| V10744 | 88 | ST23 clx | B1 | Ib | 1 |  |
| V9261 | 88 | ST23 clx | B1 | Ib | 1 |  |
| Ecor72 | 89 | - | B1 | Ib | 1 |  |
| U4191 | 93 | ST168 clx | A | Ib | 1 |  |
| U5033 | 93 | ST168 clx | A | Ib | 1 |  |
| E10077 | 162 | ST469 clx | AxB1 | Ib | 0 |  |
| V9343 | 216 | - | ABD | Ib | 0 | ΔyieJ |
| St4723 | 297 | - |  | Ib | 0 |  |
| E174 | 348 | ST156 clx | B1 | Ib | 1 |  |
| U4417 | 398 | ST398 clx |  | Ib | 0 | Δ(bglG-yieI)::IS1 |
| E294 | 399 | ST399 clx |  | Ib | 1 |  |
| U2183 | 453 | ST86 clx | B1 | Ib | 1 | ΔyieJ |
| U3104 | 533 | - | B1 | Ib | 1 |  |
| E10087 | 535 | - |  | Ib | 1 |  |
| E10092 | 536 | ST399 clx |  | Ib | 1 |  |
| F742 | 539 | - | B1 | Ib | 1 |  |
| F775 | 540 | - | AxB1 | Ib | 1 |  |
| E10086 | 541 | ST522 clx | A | Ib | 1 | ΔyieJ |
| W8987 | 542 | - | ABD | Ib | 1 |  |
| Ecor53 | 12 | ST12 clx | B2 | II | 2 |  |
| Ecor60 | 12 | ST12 clx | B2 | II | 2 |  |
| F911 | 12 | ST12 clx | B2 | II | 0 | Δ(bglF-yieH) ::IS1294 |
| J96 | 12 | ST12 clx | B2 | II | 2 |  |
| E176 | 73 | ST73 clx | B2 | II | 1 |  |
| E182 | 73 | ST73 clx | B2 | II | 2 |  |
| E471 | 73 | ST73 clx | B2 | II | 1 |  |
| Ecor51 | 73 | ST73 clx | B2 | II | 2 |  |
| Ecor52 | 73 | ST73 clx | B2 | II | 2 |  |
| Ecor54 | 73 | ST73 clx | B2 | II | 2 |  |
| Ecor56 | 73 | ST73 clx | B2 | II | 1 |  |
| Ecor57 | 73 | ST73 clx | B2 | II | 2 |  |
| F1 | 73 | ST73 clx | B2 | II | 2 |  |
| F385 | 73 | ST73 clx | B2 | II | 2 |  |
| i484 | 73 | ST73 clx | B2 | II | 2 |  |
| U2388 | 73 | ST73 clx | B2 | II | 2 |  |
| U3145 | 73 | ST73 clx | B2 | II | 1 |  |
| U3362 | 73 | ST73 clx | B2 | II | 2 |  |
| W7483 | 73 | ST73 clx | B2 | II | 2 |  |
| Ecor55 | 74 | ST73 clx | B2 | II | 2 |  |
| Ecor59 | 76 | - | B2 | II | 1 |  |
| Ecor61 | 78 | - | B2 | II | 1 |  |
| Ecor62 | 79 | - | B2 | II | 1 |  |
| Ecor63 | 80 | ST568 clx | B2 | II | 2 |  |
| Ecor64 | 81 | ST14 clx | B2 | II | 1 |  |
| Ecor65 | 82 | - | B2 | II | 1 |  |
| Ecor66 | 83 | - | B2 | II | 2 |  |
| 536 | 92 | - | B2 | II | 2 |  |
| E165 | 95 | ST95 clx | B2 | II | 1 |  |
| E175 | 95 | ST95 clx | B2 | II | 2 |  |
| E177 | 95 | ST95 clx | B2 | II | 1 |  |
| E178 | 95 | ST95 clx | B2 | II | 1 |  |
| E452 | 95 | ST95 clx | B2 | II | 2 |  |
| E457 | 95 | ST95 clx | B2 | II | 1 |  |
| U3407 | 95 | ST95 clx | B2 | II | 1 |  |
| U3454 | 95 | ST95 clx | B2 | II | 1 |  |
| E10094 | 126 | - | B2 | II | 1 |  |
| U2873 | 127 | - | B2 | II | 2 |  |
| U4437 | 127 | - | B2 | II | 2 |  |
| E10091 | 141 | - | B2 | II | 2 |  |
| St5119 | 141 | - | B2 | II | 1 |  |
| E478 | 428 | - | B2 | II | 1 |  |
| E10079 | 534 | ST95 clx | B2 | II | 2 |  |
| E475 | 537 | ST14 clx | B2 | II | 0 |  |
| E7370 | 538 | ST538 clx | B2 | II | 2 |  |
| F560 | 544 | ST12 clx | B2 | II | 2 |  |
| E422 | 547 | - | B2 | II | 1 | bglK-yieI::IS1397 |
| E464 | 550 | ST14 clx | B2 | II | 1 |  |
| F905 | 10 | ST10 clx | A | III* | 0 |  |
| E10084 | 38 | ST38 clx | D | III* | 0 |  |
| E424 | 38 | ST38 clx | D | III* | 0 |  |
| E460 | 38 | ST38 clx | D | III* | 0 |  |
| St5679 | 38 | ST38 clx | D | III* | 0 |  |
| Ecor35 | 59 | ST59 clx | ABD | III | 0 |  |
| Ecor36 | 60 | - | ABD | III | 0 |  |
| Ecor37 | 61 | ST11 clx | D | III | 0 |  |
| Ecor38 | 62 | - | ABD | III | 0 |  |
| Ecor39 | 62 | - | ABD | III | 0 |  |
| Ecor40 | 62 | - | ABD | III | 0 |  |
| Ecor41 | 62 | - | ABD | III | 0 |  |
| F645 | 62 | - | ABD | III | 0 |  |
| Ecor42 | 64 | - | ABD | III | 0 |  |
| Ecor43 | 65 | - | ABD | III | 0 |  |
| Ecor44 | 66 | - | D | III | 0 |  |
| E472 | 68 | - | D | III* | 0 |  |
| Ecor46 | 68 | - | D | III | 0 |  |
| E10098 | 69 | ST69 clx | D | III* | 0 |  |
| Ecor47 | 69 | ST69 clx | D | III | 0 |  |
| U5070 | 69 | ST69 clx | D | III* | 0 |  |
| Ecor48 | 70 | - | D | III | 0 |  |
| Ecor49 | 71 | - | D | III | 3 |  |
| Ecor50 | 72 | ST405 clx | D | III | 0 |  |
| U3292 | 130 | ST31 clx | D | III* | 0 |  |
| E179 | 350 | ST350 clx | ABD | III* | 0 |  |
| E173 | 393 | ST31 clx | D | III* | 0 |  |
| U4409 | 393 | ST31 clx | D | III* | 0 |  |
| E10093 | 405 | ST405 clx | D | III* | 0 |  |
| E10100 | 405 | ST405 clx | D | III* | 0 |  |
| E10089 | 543 | - | ABD | III* | 0 |  |
| W7716 | 545 | - | D | III* | 0 | Δ(Z5214-Z5211)::IS1 |
| E10095 | 549 | - | D | III* | 0 |  |
| Z205 | 125 | - |  | IV | 0 |  |
| RL325/96 | 133 | - |  | IV | 0 |  |
| E10083 | 546 | - |  | IV* | 0 |  |
| *E. albertii* 10457 | 383 | - |  | V | 0 |  |
| *E. albertii* 19982 | 383 | - |  | V | 0 |  |
| *E. albertii* 9194 | 383 | - |  | V | 0 |  |

(a) Sequence types (ST) are documented in the *E. coli* MLST database (http://www.ucc.ie/mlst)

(b) Strains in which absence of *bgl* genes was analyzed by Southern hybridizations are marked with an asterisk (*).

(c) Bgl phenotypes are indicated as follows: ‘0’, no functional *bgl* operon present; ‘1’, cryptic operon, which can be activated by spontaneous mutations, and ‘2’, *bgl* operon is weakly expressed. Strain ECOR49 is weakly Bgl-positive, although this strain lacks a *bgl* operon.

(d) Indels (insertion and deletions) were characterized by PCR and sequencing.
