# Supplementary material for: Fate of the H-NS–Repressed bgl Operon in Evolution of Escherichia coli
Source: PLoS Genet. 2009 Mar 6;5(3):e1000405. doi: 10.1371/journal.pgen.1000405 (PMC2646131; doi:10.1371/journal.pgen.1000405)
Supplement: Table S2 — Genome sequences used. (0.05 MB DOC) [file pgen.1000405.s007.doc]

| **Table S2: Genome sequences used** | | |
| --- | --- | --- |
| **Strain** | **Accession** | **RefSeq** |
| E. coli K12 MG1655 | U00096 | NC_000913 |
| E. coli CFT073 | AE014075 | NC_004431 |
| E. coli UTI89 | CP000243 | NC_007946 |
| E. coli K12 W3110 | AP009048 | AC_000091 |
| E. coli 536 | CP000247 | NC_008253 |
| E. coli HS | NZ_AAJY00000000 |  |
| E. coli 101-1 | NZ_AAMK00000000 |  |
| E. coli 53638 | NZ_AAKB00000000 |  |
| E. coli B171 | NZ_AAJX00000000 |  |
| E. coli B7A | NZ_AAJT00000000 |  |
| E. coli E110019 | NZ_AAJW00000000 |  |
| E. coli E22 | NZ_AAJV00000000 |  |
| E. coli E24377A | NZ_AAJZ00000000 |  |
| E. coli F11 | NZ_AAJU00000000 |  |
| E. coli APEC 01 | NC_008563.1 | NC_008563 |
| E. coli EDL933 | AE005174 | NC_002655 |
| Shigella flexneri 2a 2457T | AE014073 | NC_004741 |
| Shigella flexneri 2a 301 | AE005674 | NC_004337 |
| Shigella dysenteriae Sd197 | CP000034 | NC_007606 |
| E. albertii TW07627 | NZ_ABKX00000000 |  |
| Klebsiella pneumoniae subsp.Pneumoniae MGH 78578 | CP000647.1 | NC_009648.1 |
| Enterobacter sp. 638 | CP000653 | NC_009436 |
| Erwinia carotovora subsp. atroseptica SCRI1043 | BX950851.1 | NC_004547.2 |
