# Supplementary material for: Fate of the H-NS–Repressed bgl Operon in Evolution of Escherichia coli
Source: PLoS Genet. 2009 Mar 6;5(3):e1000405. doi: 10.1371/journal.pgen.1000405 (PMC2646131; doi:10.1371/journal.pgen.1000405)
Supplement: Table S3 — Homologs of the E. coli bgl-yieIJ locus. (0.08 MB DOC) [file pgen.1000405.s008.doc]

| **Table S3: Homologs of the *E. coli* *bgl-yieIJ* locus^a^** | | |  |
| --- | --- | --- | --- |
| **Protein** | **Accession** | **Species** | **Identity** |
| **BglG** | **P11989** | *bgl* operon antiterminator |  |
|  | A6TBM6 | Klebsiella pneumoniae subsp. pneumoniae | 62 |
|  | A4WCH6 | Enterobacter sp. (strain 638) | 62 |
|  | Q6D619 | Erwinia carotovora subsp. atroseptica | 53 |
|  | P39805 | Bacillus subtilis | 42 |
|  | A6LXA2 | Clostridium acetobutylicum | 43 |
|  | Q7N8X9 | Photorhabdus luminescens subsp. laumondii | 35 |
| **BglF** | **P08722** | PTS system β-glucoside-specific EIIBCA |  |
|  | A4WCH5 | Enterobacter sp. (strain 638) | 56 |
|  | A6TBM5 | Klebsiella pneumoniae subsp. pneumoniae | 57 |
|  | Q6D618 | Erwinia carotovora subsp. atroseptica | 57 |
|  | Q8YAT6 | Listeria monocytogenes | 45 |
|  | A7GLW4 | Bacillus cereus subsp. cytotoxis NVH 391-98 | 44 |
|  | Q97J79 | Clostridium acetobutylicum | 43 |
|  | Q7N8Y0 | Photorhabdus luminescens subsp. laumondii | 51 |
| **BglB** | **P11988** | phospho-β,D-glucosidase |  |
|  | A4WCH4 | Enterobacter sp. (strain 638) | 76 |
|  | A6TBM4 | Klebsiella pneumoniae subsp. pneumoniae | 75 |
|  | Q6D617 | Erwinia carotovora subsp. atroseptica | 72 |
|  | Q7N8Y1 | Photorhabdus luminescens subsp. laumondii | 67 |
|  | A6ATA3 | Vibrio harveyi | 66 |
|  | P40740 | Bacillus subtilis | 63 |
|  | A6LXA0 | Clostridium acetobutylicum | 59 |
|  | A4QHM9 | Corynebacterium glutamicum | 58 |
|  | Q723Y8 | Listeria monocytogenes | 58 |
| **BglH** | **P26218** | β-glucoside specific porin |  |
|  | A6TG27 | Klebsiella pneumoniae subsp. pneumoniae | 77 |
|  | Q6D616 | Erwinia carotovora subsp. atroseptica | 53 |
| **BglI-YieK** | **P31470** | Uncharacterized protein |  |
|  | A6TG26 | Klebsiella pneumoniae subsp. pneumoniae | 71 |
|  | Q6D615 | Erwinia carotovora subsp. atroseptica | 70 |
|  | A7MMY5 | Enterobacter sakazakii | 66 |
| **BglK-YieL** | **P31471** | Uncharacterized protein |  |
|  | A6TG25 | Klebsiella pneumoniae subsp. pneumoniae | 73 |
|  | Q838J6 | Enterococcus faecalis | 50 |
|  | Q4ETM3 | Listeria monocytogenes str. 1/2a F6854 | 51 |
| **YieJ-CbrC** | **P31469** | Uncharacterized protein |  |
|  | A7UG98 | Escherichia albertii | 89 |
|  | A8RVW1 | Clostridium bolteae ATCC BAA-613 | 49 |
|  | A8FGE4 | Bacillus pumilus (strain SAFR-032) | 45 |
| **YieI-CbrB** | **P31468** | Uncharacterized protein |  |
|  | A4W5K2 | Enterobacter sp. 638 | 33 |
|  | Q57T97 | Salmonella choleraesuis | 31 |

^a^ Homologs of the *E. coli* encoded proteins indicated in the first column were identified by BLAST using the UNIPROT database. For each species one representative with the highest identity was listed.
