# Supplementary material for: Fate of the H-NS–Repressed bgl Operon in Evolution of Escherichia coli
Source: PLoS Genet. 2009 Mar 6;5(3):e1000405. doi: 10.1371/journal.pgen.1000405 (PMC2646131; doi:10.1371/journal.pgen.1000405)
Supplement: Table S4 — Oligonucleotides used for MLST and bgl-Z typing. (0.12 MB DOC) [file pgen.1000405.s009.doc]

| **Table S4: Oligonucleotides used for MLST and *bgl*-Z typing** | | |
| --- | --- | --- |
| **name** | **sequence (5’ to 3’)** | **description^a^** |
| S4 | GGATGGACATTGACGAAGC | phoU: 443 to 461 |
| S5 | GGATTGTTACTGCATTCGC | bgl: +42 to +60 |
| S32 | CCACTGCGGCAAGCTGAG | yieJ: 549 to 566 |
| S34 | GGATAAACTGCTGGCGGG | phoU: 690 to 707 |
| S54 | CCTCTAGAATTCCGCGCCCCATGACGA | bgl: +224 to +205 |
| S91 | GTGATTTGCATGTTCATAGCAAGGAC | bgl: +148 to +123 |
| S92 | CAAGAGGAATATGACTTAAGAGTTCG | bgl: +342 to +317 |
| S145 | CCGGTCGACGCGTTCGCGCGGATGGACATTGACGAAGC | phoU: 431 to 461 |
| S157 | CCCGTCGACttataactgcgagcatggtca | bgl: -76 to -55 |
| S186 | ATAACCAGAGAATACTGGTGAAGTCGGGT | bgl: +75 to +103 |
| S201 | GCGTCTCTAGAAATATTTCAGTGTTCTTTGCGCACG | bgl: +974 to +950 |
| S212 | CCTCTAGATTTTTATAACGAACATCCAGGTTCG | bgl: +25 to +1 |
| S220 | GCGGATCCATGAACATGCAAATCACCAAAATTCTCA | bgl: +132 to +159 |
| S224 | GGGGATCCACCCGCAAGCATGGCAATGT | bgl: +841 to +860 |
| S252 | AGATGCTGCACGACGTGCTGG | phoU: 410 to 430 |
| S253 | GTCACGGCAAACGAAAGCGC | bgl: +5427 to +5446 |
| S257 | GCTCTAGATGCCCTCTACCGCTTTGCG | bgl: +1097 to +1079 |
| S309 | CCGTCTAGACATCGATTTTTATCACCGT | bgl: +3171 to +3189 |
| S312 | TGATCCCCGCCTGCGC | bgl: +3352 to +3337 |
| S335 | CTTCAGGATCGAGCGTAATACCA | yieH: 121 to 99 |
| S336 | CGCGGAAAATCGTCAGTAACA | yieG: 394 to 374 |
| S346 | CCACTCTGATTTCGAATCTATTCGT | bgl: +5777 to +5801 |
| S347 | GCACAATAAGCCGATCGTTCA | yieH: 567 to 587 |
| S348 | CGGGTCACAGAAACGTTATCGT | CFT073 yieI: 219 to 240 |
| S349 | GTAACGACATGTTGATTTCATTAAACGT | bgl: +3529 to +3502 |
| S350 | GCTGCCGCAGAATTAGCACT | bgl: +8002 to +8021 |
| S351 | GGATGATGCCAGCATAGAAGGT | yieJ: 222 to 243 |
| S352 | GAGCGGCATAACCTGAATCTGA | IS1: 625-646 |
| S373 | CCGGTCGACGCTGCCAGAATATTTGTGAGTTTATCT | phoU: 613 to 640 |
| S406 | GCGGATCCGCAAAGCGGTAGAGGGC | bgl: +1078 to +1096 |
| S407 | TGATGATAAAGGTAATCTGCTAAACCG | bgl: +1371 to +1397 |
| S408 | GCCTGGTTGTGCAGCATTCTG | bgl: +1771 to +1791 |
| S409 | GATCAGCGGCGTTGACGAG | bgl: +2171 to +2189 |
| S410 | CATTCACGTCGCTGATACCACG | bgl: +2571 to +2592 |
| S411 | GCGGATCCTTTTATCGTTAGCGAATGATGG | bgl: +2990 to +2966 |
| S412 | CCATGCGGCAAAAGCGATGAATGT | yieH: 447 to 470 |
| S413 | CCGATCGTTCACCCGAAAGTCACCA | yieH: 557 to 601 |
| S429 | GGCGAAAAACTTGCTGATAATTGT | bgl: +7860 to +7883 |
| S437 | CGACGGTACGCTGGTCGA | yieH: 33 to 50 |
| S460 | CTTCGGTAACCGGACCTTGC | bgl: +7942 to +7923 |
| S463 | AGTGCCTGACAGCTACGTGACG | bgl: +7815 to +7836 |
| S467 | CAATCCTTTACTCAGTAAGCTTAACCGAGTGCTAATTCTGC | bgl: +8033 to +8008 |
| S479 | CCGTCGACCCACCAGCAAATGAGCCGTGTCGCGG | bgl: +7337 to +7354 |
| S536 | CTGAATGCTAAAGCGGCAGATC | bgl: +6848 to +6869 |
| S537 | CAGTGGCTTGGGATGATATTTGA | yieJ: 32 to 54 |
| S547 | CGCTTAGTTTTTCATTATCATTAGGGA | Z5214: 481 to 455 |
| S548 | GTCGATTGTGATGATAAAATACGTTCT | Z5211: 2248 to 2274 |
| S560 | GCGAAGGAAGCCTCACAAGA | bgl: +4304 to 4323 |
| S561 | CCCGTTATTATCCTGATTATCTTTTTC | bgl: +5477 to +5451 |
| S562 | GTCGTTACACGCGCCATTCAC | bgl: +3522 to 3542 |
| S563 | CGGGTGAATATTGTCCGGAAC | bgl: +6346 to +6326 |
| S564 | CCGTAGCGCTTAGACATTTGTGA | bgl: +4273 to 4252 |
| S565 | GCGTTCCAGAATGCTGCACA | bgl: +1797 to 1778 |
| S566 | GCTGGTTCGGTGATACCAAACA | bgl: +2213 to 2192 |
| S586 | GGTTCTTTGGGTGATAATACATCCA | yieI: 111 to 135 |
| S587 | CTTTTGGTAATAATACAGGTACTTCCATTGT | CFT073 yieI: 115 to 145 |
| S588 | CCGGTTACCGAAGATGTTCCA | bgl: +7930 to +7950 |
| S589 | CATTTTTGTGGCAATCTGCCA | CFT073  bgl: +7692 to +7712 |
| S712 | ATTCTGCTTGGCGCTCCGGG | MLST adk |
| S713 | CCGTCAACTTTCGCGTATTT | MLST adk |
| S715 | GTACGCAGCGAAAAAGATTC | MLST fumC |
| S716 | TCGGCGACACGGATGACGGC | MLST gyrB |
| S717 | GTCCATGTAGGCGTTCAGGG | MLST gyrB |
| S718 | ATGGAAAGTAAAGTAGTTGTTCCGGCACA | MLST icd |
| S719 | GGACGCAGCAGGATCTGTT | MLST icd |
| S720 | ATGAAAGTCGCAGTCCTCGGCGCTGCTGGCGG | MLST mdh |
| S721 | TTAACGAACTCCTGCCCCAGAGCGATATCTTTCTT | MLST mdh |
| S722 | TCGGTAACGGTGTTGTGCTG | MLST purA |
| S723 | CATACGGTAAGCCACGCAGA | MLST purA |
| S724 | AGCGTGAAGGTAAAACCTGTG | MLST recA |
| S725 | ACCTTTGTAGCTGTACCACG | MLST recA |
| S727 | TCACAGGTCGCCAGCGCTTC | MLST fumC |
| S733 | CGGATGTGTGAATTACGCTCCGG | bglK to yieJ/I intergenic region |
| S734 | CTCCTGAACACAATATTTATTCGCCCG | Z5214 |
| S735 | AAAGCACTATCAAACCAACTGGAACATATAAAAT | Z5211 |
| S766 | CGCATTCGCTTTACCCTGACC | MLST recA |
| S767 | TCGTCGAAATCTACGGACCGGA | MLST recA |
| S776 | GCCTTTCTTGAGGCAATCGCCTG | MLST adk |
| S777 | CAACTTGTTGATAATTGTAGCGGAAAAGTG | MLST adk |
| S778 | CAGGTAATGACTGCCAGTTCATCTGC | MLST fumC |
| S782 | TCGAACCAATCCAGAATATTA | Z5211 |
| S785 | TACGGACTGCCCGTTGACGG | IS629 |
| S786 | CCAGGTAATGATTTACAGCGGCAAG | IS1397 |
| S787 | TCCGGTGCATTTGCAATTAACTG | Z5211 |
| S788 | GCATCCGGCAATGTGTCCAG | E. albertii phoU to yieJ |
| S789 | TTCCACGAGCAGACAGGACGTT | E. albertii yieJ to yieH intergenic region |

a. Coordinates prefixed with ‘+’ refer to positions relative to transcription start of the *bgl* operon in *E. coli* K12. All other coordinates refer to positions relative to translational start sites of the respective genes in *E. coli* K12 MG1655 and O157:H7 EDL933, respectively, or *E. coli* strain CFTO73, where indicated.
